# Reliable charge assessment on encapsulated fragment for endohedral systems

**DOI:** 10.1038/s41598-018-21240-0

**Published:** 2018-02-13

**Authors:** A. J. Stasyuk, M. Solà, A. A. Voityuk

**Affiliations:** 1 Institut de Química Computacional and Departament de Química, Universitat de Girona, C/ Maria Aurèlia Capmany 69, 17003 Girona, Catalonia Spain; 2 Institució Catalana de Recerca i Estudis Avancats, 08010 Barcelona, Spain

## Abstract

A simple scheme to determine charge distribution in endohedral complexes is suggested. It is based on comparison of inner-shell atomic orbital energies of the encapsulated species to the corresponding energies in reference systems with unambiguously defined charges on X. This robust approach is applied to endohedral borospherenes X@B_39_, for which the conventional schemes provide in some cases quite different results. Efficiency of proposed scheme also has been proven for typical fullerene based Sc_3_N@C_80_ endohedral complex.

## Introduction

Atomic charge is one of the most widely used concepts in chemistry. But, it can unambiguously be defined only in some trivial cases. For instance, in homogeneous diatomic molecules with the total charge Q, the charge on each atom is equal to Q/2. In general, however, some assumptions should be made in order to quantify the charge distribution in a molecular system. Many different schemes to define atomic charges have been developed. Most popular of them are schemes based on the distribution of the electron density obtained from quantum-mechanical calculation and its subsequent transformation into atomic charges –Mulliken^1^, Lowdin^2^, Hirshfeld^3^, CM5^4^, Weinhold^5,6^ (natural population analysis, NPA), and Bader^7,8^ (quantum theory of atoms in molecules, QTAIM) population analysis.

Discovery of buckminsterfullerene C_60_^9^, and its La encapsulated derivative^10–12^ made a new milestone in chemistry. Fullerenes containing confined atoms or small molecules are called endohedral fullerenes (EFs). Most often the incarcerated species is a metal atom or metallic cluster, and such systems are called endohedral metallofullerenes (EMFs)^13–22^. Charge transfer between the encapsulated species and the cage in EMFs is important because it determines the most suitable cage among the many possible isomers. For instance, in Sc_2_C_2_@C_68_ with a formal charge transfer of 4 electrons, the cage corresponds to the 6073 isomer^23^, whereas the cage that encapsulates Sc_3_N@C_68_ with a formal charge of 6 electrons is the 6140^24^. Although many EMFs have been intensively studied they continue to attract much attention of researchers from various fields of natural sciences. Recent discovery by Zhai *et al*. of all-boron fullerenes B_40_^−^ and B_40_^0^ (referred in literature as borospherenes)^25^ has given a new impetus to the chemistry of endohedral compounds and stimulated a large number of experimental and theoretical studies. A few months later Bai *et al*. studied computationally the viability of endohedral metalloborospherenes M@B_40_ (M = Ca, Sr). DFT calculations revealed great stability due to the almost perfect match in size of encapsulated fragment and cage. It was found that all these metalloborospherenes are formally charge-transfer M^2+^B_40_^2−^ complexes^26^. Other endohedral complexes of borospherenes based on B_36_^−^ ^27^, B_38_^2−^ ^28^, and B_39_^−^ ^29^, cages demonstrate similar behaviour. A number of works predicting the existence of endohedral borospherenes have been published very recently^30–34^. Making an analogy with carbon fullerenes we can expect that endohedral borospherenes will be synthetized at an early date.

## Results and Discussions

Focusing our attention on endohedral Cl@B_39_ borospherene complex based on B_39_ cage subunit, we have encountered the problem of the reliability of charge determination on central fragment. The population analysis carried out within various schemes demonstrates significantly different distribution of charges. However, a reliable definition of the charge localized on the encapsulated fragment as well as its changes is important for the understanding of chemical reactivity, and, in particular, for photo-induced charge transfer (CT) process in endohedral compounds^35–38^.

In this work, we present a new scheme for assessment of charge distribution on endohedral complexes. Proposed method is based on straightforward physical model and could be used as a convenient tool for the culling of inadequate charge schemes. All calculations were performed with the PBE0/Def2-TZVP (with ECP-28 and ECP-60 for Ag and Au atoms^39^ correspondently) method^40–42^ using Gaussian 09 program^43^.

Numerous proofs that metal-encapsulating borospherenes could demonstrate remarkable charge transfer properties have been recently reported. Among them, the work published by Chen *et al.*^29^ demonstrates at first-principles level the viability of the axially chiral metalloborospherenes Ca@B_39_^+^. At the same time there are no examples of halogen encapsulated borospherenes, despite the fact that such complex is a unique system of halogen encapsulate into superhalogen unit. The uniqueness of the Cl@B_39_ complex is that both fragments exhibit high electron affinity (Cl – 3.612 eV^44^, B_39_ – 3.845 eV^45^). Thus it seems extremely difficult to predict charge distribution in such system. With a certain degree of confidence it can be assumed that charge separation should be observed, however it is completely unclear which of the fragments will be negatively charged and which positively.

### Charge distribution analysis for B_39_ boroshperene complexes

**Figure 1:**
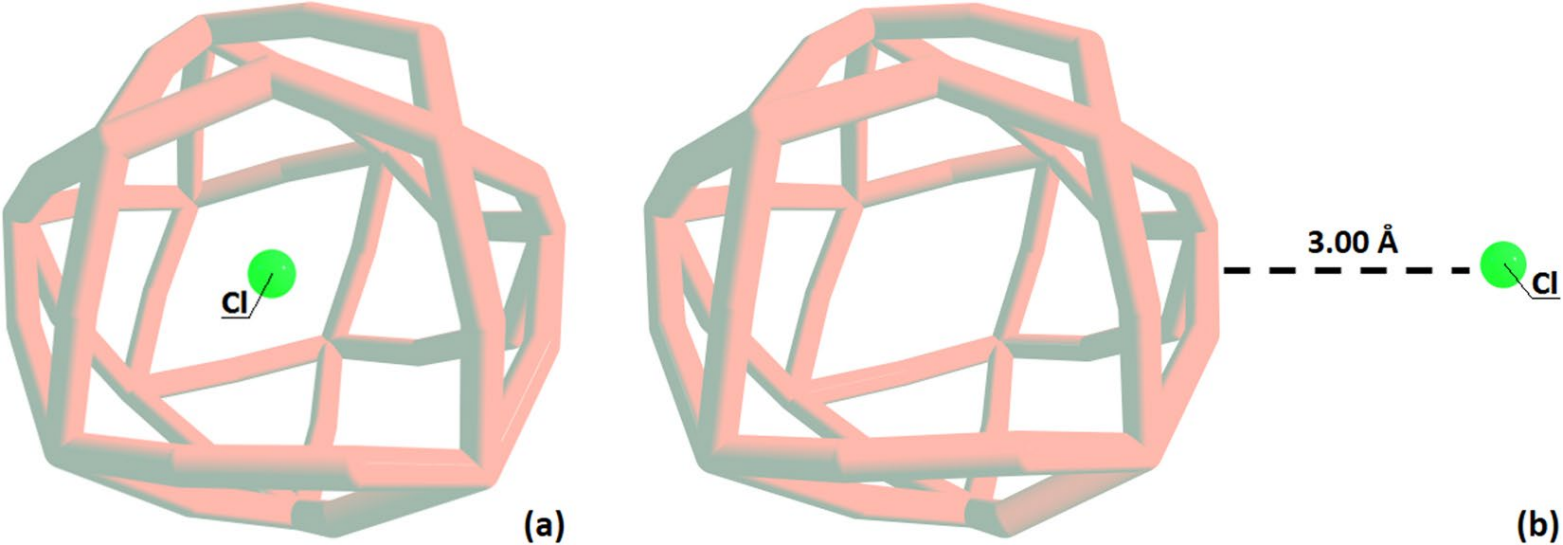
Graphical representation of endohedral boroshperene complex Cl@B_39_ (**a**) and (B_39_ + Cl) vdW complex (**b**).

We focused our attention on complexes such as Cl@B_39_ (Fig. 1a), in particular on the description of charge states in such systems using various approaches. We found that charges on internal fragment, as well as on the cage, depend dramatically on the population scheme used.

In contrast, for van der Waals complex (Fig. 1b) composed of B_39_ and Cl separated by 3 Å, (B_39_ + Cl) vdW, the predicted charges demonstrate a significant similarity (Table 1).

**Table 1:** Charge density analysis (in electrons) performed with Mulliken, Löwdin, Hirshfeld, CM5, QTAIM, and Natural Population Analysis (NPA) schemes for endohedral Cl@B_39_ and (B_39_ + Cl) van der Waals (vdW) complex obtained at PBE0/Def2-TZVP level of theory. Total charge is in all cases zero.

	Mulliken	Löwdin	Hirshfeld	CM5	QTAIM	NPA
	Cl@B_39_					
B_39_	0.111	−0.758	−0.002	−0.114	0.658	0.560
Cl	−0.111	0.758	0.002	0.114	−0.618	−0.560
	(B_39_ + Cl) vdW					
B_39_	0.410	0.239	0.393	0.385	0.580	0.480
Cl	−0.410	−0.239	−0.393	−0.385	−0.547	−0.480

As can be seen from Table 1, for Cl@B_39_ complex, charge on the Cl fragment can vary from −0.62 e (QTAIM) to 0.76 e (Löwdin) depending on the used scheme. At the same time, charge on the cage ranges from −0.76 e (Löwdin) to 0.66 e (QTAIM). Presuming that behaviour of these fragments in endohedral complex does not change significantly, the reliability of the charges obtained based on Löwdin population analysis (*Q*_*Cl*_ = 0.76), as well as on Hirshfeld (*Q*_*Cl*_ = 0.00) and CM5 (*Q*_*Cl*_ = 0.14) schemes should be called into question. For the van de Waals complex, the obtained charge values on the Cl atom are much closer to each other and vary from −0.24 e (Löwdin) to −0.58 e (QTAIM).

The main problem in this case is the selection of the most reliable method/scheme of charge assessment, which is almost impossible without resorting to chemical intuition.

### Model description

It is well known that the ionization potential (binding energy) of the inner-shell electrons of an atom in a molecular system depends on the charge state and chemical surroundings of the atom^46^. A linear relationship of the binding energy (E_b_) measured by X-ray photoelectron spectroscopy (XPS) and an atomic charge *q* computed with quantum mechanical methods (Eq. (1)) was established for different classes of chemical compounds

1 $${\rm{\Delta }}{E}_{b}={E}_{b}-{E}_{b0}\approx Aq$$

The XPS shift *ΔE*_*b*_ is determined with respect  to the binding energy *E*_*b0*_ found for a reference. Further studies showed, however, that the electrostatic potential *φ* created by chemical surroundings has to be accounted for^47^

2 $${\rm{\Delta }}{E}_{b}=Aq+\phi $$

There are inherent physical limitations that prevent accurate determination of the atomic charges. In particular, the parameters *q* and *φ* in Eq. (2) refer to the ground electronic state of the system, while the measured XPS shift, ΔE_b_, reflects both the ground and the final excited state (with a hole in the inner shell). To separate the contributions *Aq* and *φ*, some additional assumptions should be used. In spite of these and some other restrictions, XPS has been widely employed for chemical analysis^48^.

**Figure 2:**
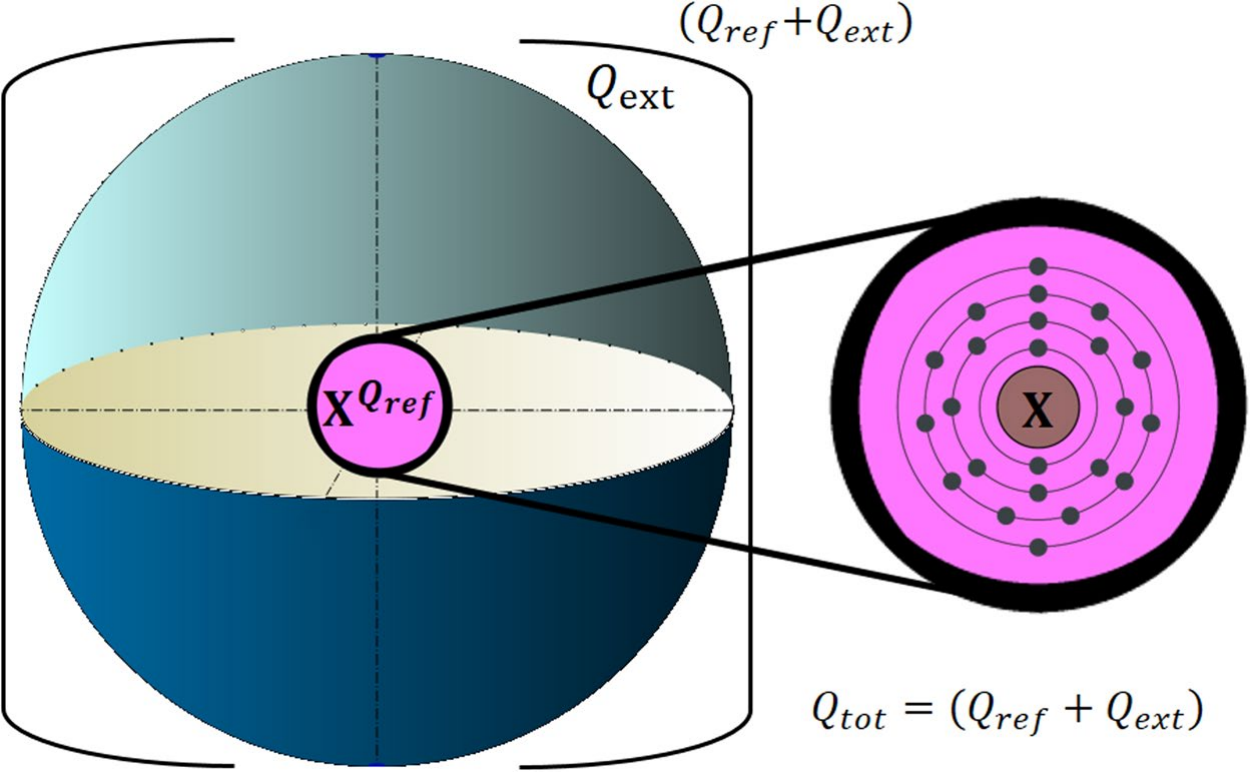
Physical model used in this work.

Let’s consider a model system consisting of a multi-electron fragment X with partial charge Q_ref_ incorporated into a cage constructed of N atoms with partial charge Q_ext_ (Fig. 2).

Similar to Eq. (2) we assume that the orbital energy of core electrons of an atom of fragment X (X consists of either a single atom or several atoms) can be approximately presented as a simple function of the charge Q_X_ *on fragment* X, corrected by the external electrostatic potential φ_ex_ on atom due to the cage

3 $${\varepsilon }_{k}^{corr}({Q}_{x},{\phi }_{ext})={\varepsilon }_{k}({Q}_{x})+{\phi }_{ext}$$

The non-trivial point here is how to calibrate ${\varepsilon }_{k}^{corr}({Q}_{x},{\phi }_{ex})$ so that the charge Q_X_ in the reference systems is uniquely defined.

We propose the following step-by-step scheme to derive the charge on X in X@Cage species:

1. One constructs reference systems where charge on X is unambiguously defined. The reference systems for such a situation include the fragment X carrying the total charge Q_ref_ (e.g. Q_ref_ = −1, 0, + 1 *etc*) embedded into a sphere having the charge Q_ext_. The total charge Q_tot_ of the reference system, Q_tot_ = Q_ref_ + Q_ext_, is equal to the charge of X@Cage. In most cases X@Cage is neutral (Q_tot_ = 0) and thus for any reference system Q_ext_ = −Q_ref_. If X@Cage is charged, Q_ext_ = Q_tot_ − Q_X_. The radius of the sphere R is determined by the cage geometry. For a cage comprising N atoms, R can be defined as
  4 $$\frac{1}{R}=\frac{1}{N}\sum _{i=1}^{N}\frac{1}{{r}_{i}}$$
  where *r*_*i*_ is the distance from atom *i* in the cage to the center of the cage determined by the non-*weighted* mean of the coordinates of the atoms in the cage. In this approach:
  5 $${\phi }_{ext}=\frac{{Q}_{ext}}{R}$$
2. Quantum mechanical calculation with molecular charge Q_ref_ (e.g. Q_ref_ = −1, 0, + 1, *etc*.) are carried out. The computed orbital energy of inner shell *K*, ${\varepsilon }_{K}({Q}_{ref})$, together with the electrostatic potential created by the sphere provides ${\varepsilon }_{K}^{corr}({Q}_{ref})$:
  6 $${\varepsilon }_{K}^{corr}({Q}_{ref})={\varepsilon }_{K}({Q}_{ref})+\frac{{Q}_{ext}}{R}$$
  The found values ${\varepsilon }_{K}^{corr}$ are used to obtain a quadratic interpolation of the ${\varepsilon }_{K}^{corr}({Q}_{ref})$ dependence. Note that the encapsulated species X comprise usually only several atoms and its calculation is very fast.
3. Quantum mechanical calculations of X@Cage provide the ${\varepsilon }_{K}$ value in the “real” system. Using the interpolated function ${\varepsilon }_{K}^{corr}({Q}_{ref})$ derived in the previous step we could directly obtain the charge Q_X_ on the encapsulated fragment.

These three steps represent the simplest scheme to estimate charge separation between encapsulated species X and the cage in endohedral fullerenes and in similar structures. As shown below, a more elaborated treatment of the electrostatic embedding in the reference systems does not provide significantly different results. Also, as we will see, the proposed scheme is quite robust to choosing the inner shell *K* in X when constructing the interpolated function $\,{\varepsilon }_{k}^{corr}({Q}_{ref})$.

The classical point charge model was used to evaluate the electrostatic correction. This model implies the replacement of cage atoms by array of point charges placed at the nuclei of the constituted atoms. This approach has proven itself well in various fields of chemical science and computational biology^49,50^.

**Figure 3:**
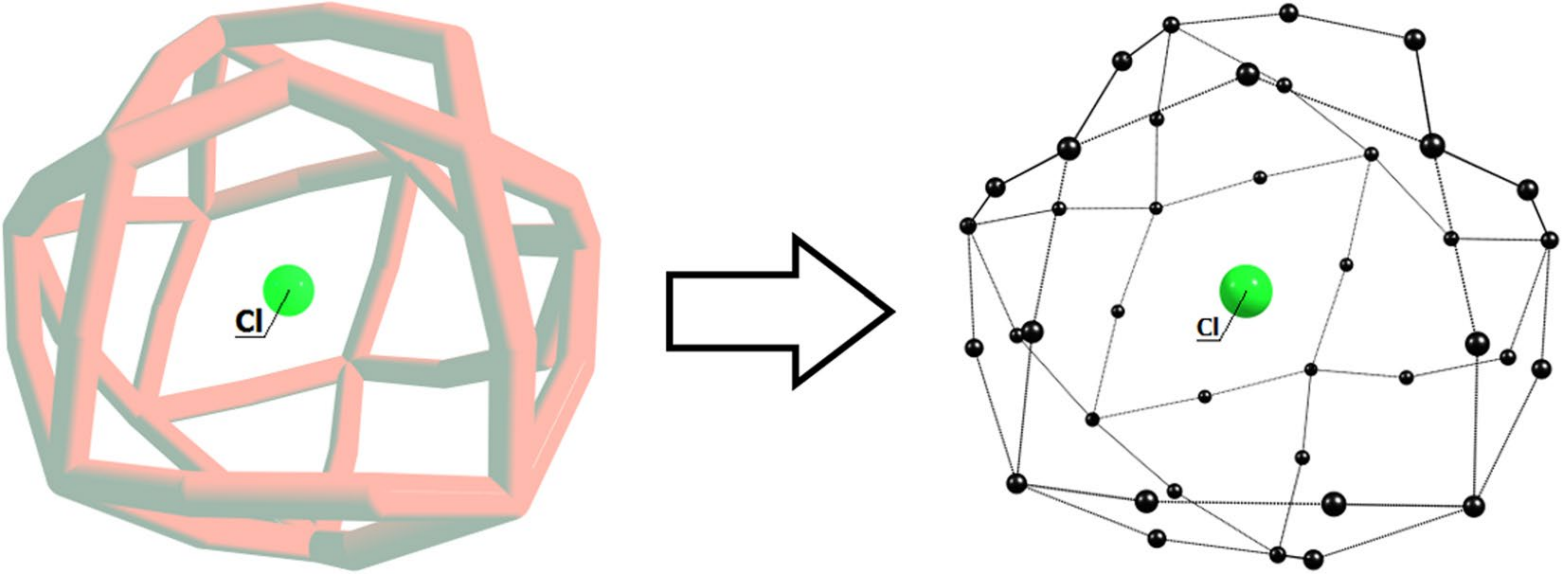
Transformation of Cl@B_39_ complex within the point charge model framework necessary for calibration curve construction. Left part of figure corresponds to Cl@B_39_ complex, right part represents central fragment (Cl) surrounded by compensation charges (black spheres) allocated at former boron nuclei places.

To verify the applicability of the proposed model to the charge assessment of the central fragment in endohedral complexes, the borospherene species Cl@B_39_ was considered in detail (Fig. 3).

Primary, it is necessary to perform a series of calculations for Cl fragment in various charge states. For this we have selected 5 states – Cl^2−^, Cl^1−^, Cl^0^, Cl^1+^, and Cl^2+^ in their ground states. Despite the fact that Cl^2+^ as well as other halogen cations goes beyond conventional chemical principles and its existence is apparently impossible under ordinary conditions, we will take it into account as a model point to have a wider range of calibration. Including a large set of charge states into calibration makes a proposed scheme more robust and universal due to the fact that even in difficult cases a result can be found within the interpolation grid. Of course, in case of Cl fragment, data about its possible charge states is well known, but in case of more sophisticated encapsulated unit a necessary information can simply not be available. Moreover, it was found that inclusion of 1s orbital energy of Cl^2+^ unit does not lead to physically meaningless result, therefore we recommend to use greater number of charge states for constructing a calibration curve.

To take into account correction to electrostatic potential, boron atoms forming cage have to be replaced with point charges (Fig. 3) in such way that total compensation charge has to be equal to the charge on Cl fragment in current charge state. For example, for Cl^1+^ cation compensation charge have to be equal to −1, while for Cl^1−^ fragment the compensation charge have to be equal to +1. It seems obvious that there are more than one way of the compensation charge distribution over cage nodes when the neutrality condition is satisfied.

To investigate the effect of compensation charge distribution on 1s orbital energy we performed calculations for 4 different models (Table S1, ESI). To construct first of them, even charge distribution over the cage were used, while for Cl^0^ charge state no compensation point charges have been applied (CCS1, Table S1). In next three schemes, the charges on cage were generated randomly to compensate the charge −1 and then be linearly scaled to the charge state −2. In the case of Cl^1+^ and Cl^2+^, compensation charges on the cage were obtained from previously generated only with opposite sign (CCS2, CCS3, and CCS4 in Table S1). Last scheme is characterized by random generation of charges on all cage nodes for each state of Cl fragment. Only equality of the charge on Cl fragment and sum of compensation charges was monitored (CCS5, Table S1). Calibration curves and equations of approximate functions are shown in Fig. S1. Detailed analysis of given charge distribution schemes revealed that electrostatic correction for 1 s orbital energy value depends only very weakly on particular compensation charge scheme. For the studied charge distributions, the range of the correction values is about one order of magnitude less than the correction values themselves (Table S2). At the same time, central fragment charges predicted on the basis of the mentioned charge compensation schemes are in the range from −0.44 e to −0.51 e. Thus, taking into account small differences in charge prediction caused by particular charge compensation method, we will use the even charge distribution as a compensation method.

**Figure 4:**
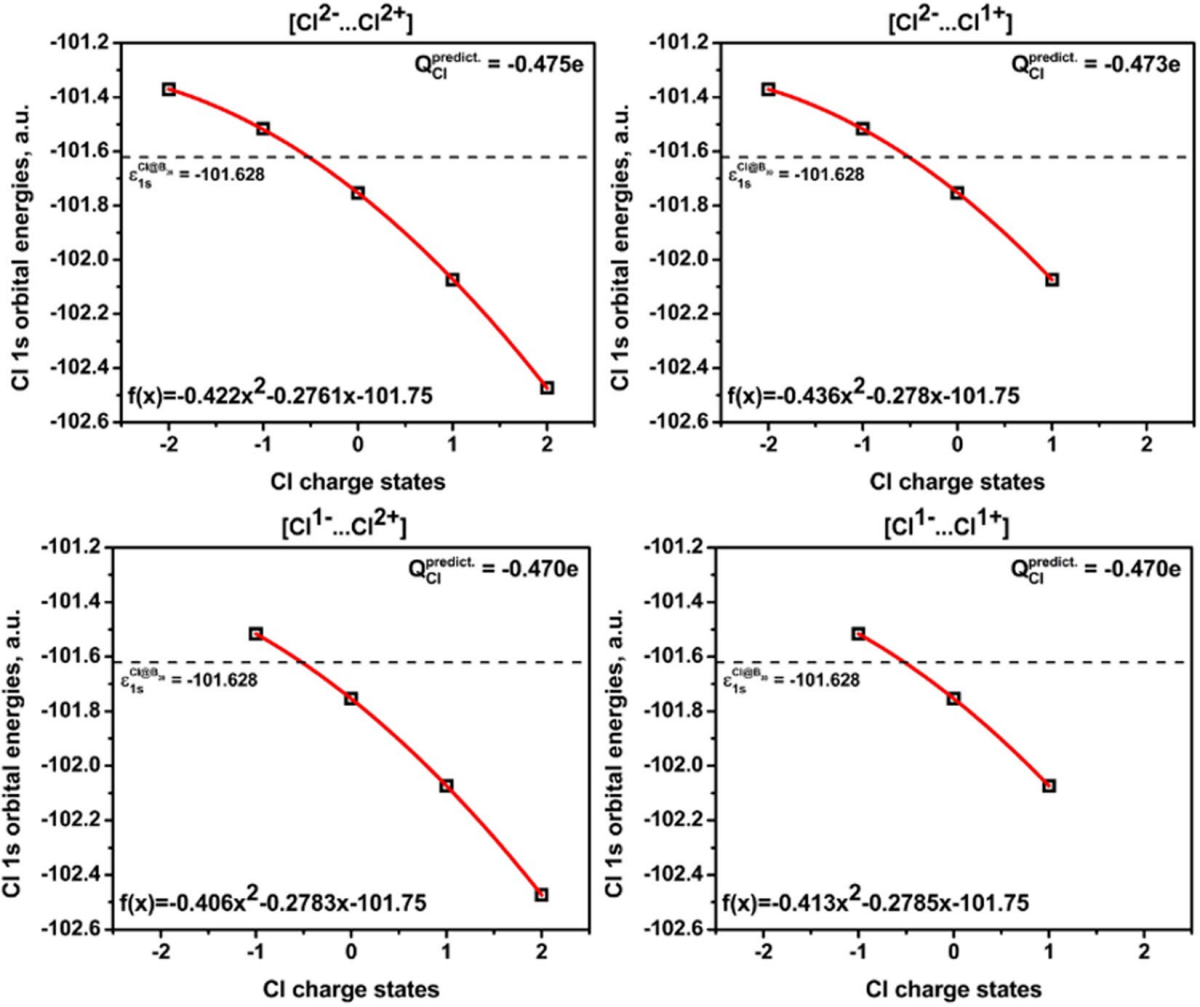
Calibration curves constructed using various number of reference points (top left – [Cl^2−^…Cl^2+^] – 5 points, top right – [Cl^2−^…Cl^1+^] – 4 points, bottom left – [Cl^1−^…Cl^2+^] – 4 points and bottom right – [Cl^1−^…Cl^1+^] – 3 points) for charge prediction on central Cl fragment in Cl@B_39_ complex (Cl 1s orbital energy in complex of interest = −101.628 a.u.).

At the next stage of our work, we studied the effect of the charge states taken into consideration on the predicted charge values of the Cl@B_39_ complex. It was found that the charge predicted within the proposed method depends weakly on charge states of Cl fragments that were taken into account in calibration. Four different calibration curves were built using various Cl charge states – 1 ^st^: [Cl^2−^…Cl^2+^] with 5 reference points (*Q*_*Cl*_ = −0.475), 2^nd^: [Cl^2−^…Cl^1+^] with 4 points (*Q*_*Cl*_ = −0.473), 3^rd^: [Cl^1−^…Cl^2+^] with 4 points (*Q*_*Cl*_ = −0.470), and 4^th^: [Cl^1−^…Cl^1+^] with 3 points (*Q*_*Cl*_ = −0.470) (Fig. 4).

As can be seen in Fig. 4, minor differences (of less than 0.01 e) in the predicted charge on the Cl fragment obtained from different calibration curves are found. This error is one order of magnitude smaller than the error caused by the use of different charge compensation schemes.

Thus, we can conclude that for studied Cl@B_39_ complex proposed method has shown its best side. However, we were wondering if proposed method can equally work well with other K-type orbitals. To check this, Cu@B_39_ complex has been considered. Cu atom has 3 inner s-orbitals. Calibrations curves construct for 1s, 2s, and 3s orbitals based on five Cu charge states [Cu^2−^…Cu^2+^] reference points predict charge on central fragment 0.416 e, 0.391 e, and 0.398 e correspondently (Fig. S2, ESI). Thus, one can assume that any inner orbital, as far as it not involved in any orbital interaction with the cage orbitals, could be used for construction of the calibration. The latest suggests that the proposed scheme can be extended to “heavy” elements, for which pseudopotentials are used.

### Model verification

Finally, to verify the proposed approach several systems with general formula X@B_39_, where X represents halogens (F, Cl, Br), small radicals (NO, CN, FO, CF) or metals of 1B group of periodic chart (Cu, Ag, Au) were investigated (Fig. S3). The summary results are presented in Table 2.

**Table 2:** Charge density analysis performed with Mulliken, Löwdin, Hirshfeld, CM5, QTAIM, and NPA schemes for endohedral X@B_39_ complex and the predicted charge of X (*Q*_x_ predicted)^a^ obtained within the method proposed in this work. Units are electrons.

X	Mulliken	Löwdin	Hirshfeld	CM5	NPA	AIM	${{\boldsymbol{Q}}}_{{\boldsymbol{X}}}\,\,$predict.^a^
F	−0.603	−0.300	−0.334	−0.305	−0.842	−0.822	−0.585 (1s F)
Cl	−0.111	0.758	0.002	0.114	−0.560	−0.618	−0.475 (1s Cl)
Br	−0.326	1.142	0.209	0.417	−0.258	−0.357	−0.251 (1s Br)
NO	0.444	0.842	0.458	0.473	0.127	0.030	0.212 (1s O) 0.253 (1s N)
CN	−0.793	0.162	−0.177	−0.117	−1.070	−0.899	−0.763 (1s N) −0.841 (1s C)
FO	−0.267	0.297	0.027	0.085	−0.532	−0.619	−0.252 (1s F) −0.356 (1s O)
CF	0.014	0.865	0.472	0.582	0.001	0.086	0.176 (1s F) 0.257 (1s C)
Cu	0.187	0.306	0.487	0.825	0.754	0.719	0.416 (1s Cu) 0.391 (2s Cu) 0.398 (3s Cu)
Ag	0.142	0.620	0.506	0.984	0.738	0.586	0.443 (4s Ag)
Au	−0.589	0.705	0.560	0.972	0.799	0.417	0.441 (5s Au)

^a^Information in brackets refer to the atoms and orbitals used for calibration.

**Figure 5:**
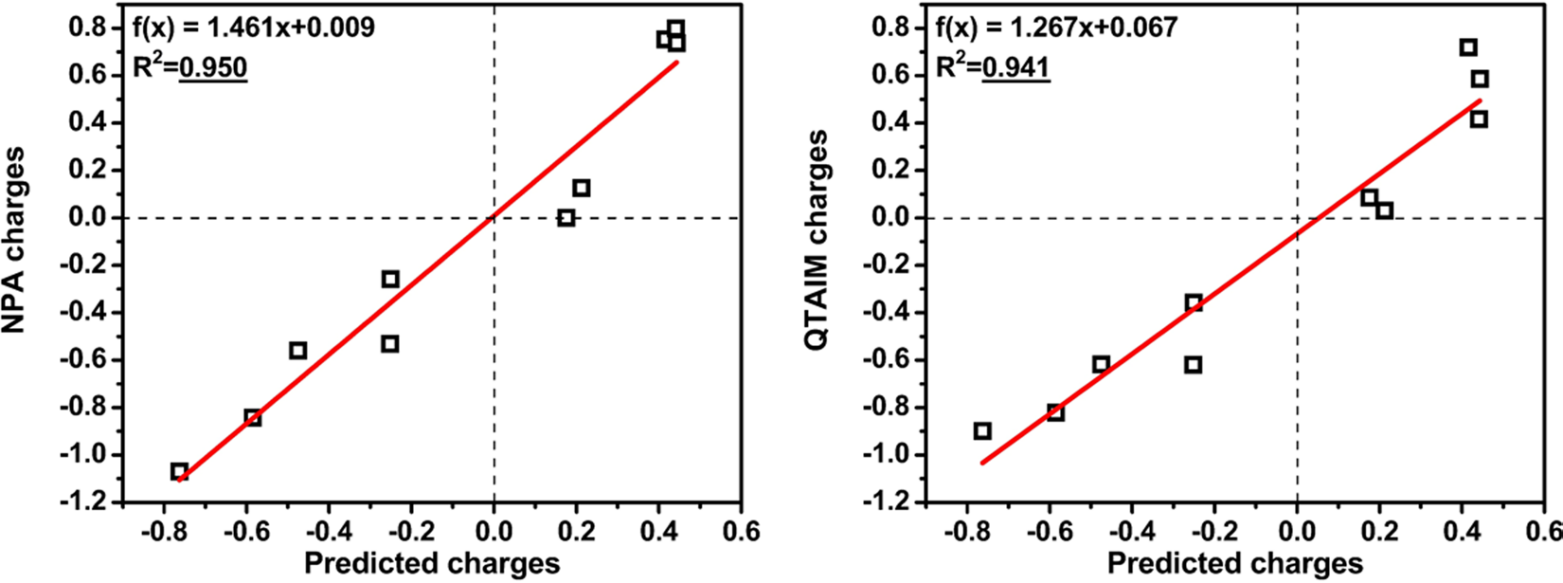
Correlations of the NPA and QTAIM charges with the results obtained by the proposed method. Units are electrons.

As can be seen from Table 2, the proposed charge assessment approach provides the physically adequate results in all studied cases. For each cases, Mulliken and Löwdin charge assessment schemes provide the worst performance with correlations with the ${Q}_{X}\,\,$predicted charges of *R*^2^ = 0.388 for Mulliken and *R*^2^ = 0.172 for Löwdin, respectively. Moreover, sometimes they provide physically meaningless results, such as in case of Au@B_39_ (Mulliken) or Cl@B_39_, Br@B_39_, FO@B_39_ and CN@B_39_ (Löwdin) complexes. Hirshfeld charges are quite well correlated with the predicted charges (*R*^2^ = 0.909) but the charges are noticeably underestimated. CM5 method yields comparable to Hirshfeld correlation with the predicted results with *R*^2^ = 0.896. Equally to Hirshfeld scheme, significant charge underestimation for CM5 is observed. In some cases Hirshfeld and CM5 schemes does not reproduce the sign of charge on the central fragment (Fig. S4, Table S3, ESI). Finally, the best correlations between calculated and predicted charges were found for NPA and QTAIM schemes (Fig. 5).

Thus, we can conclude that the predicted charge assessment method provide results comparable with well-known methods and can be used as a fast and convenient selection tool that avoids assignment of unphysical charges to encapsulated species.

To demonstrate that the proposed method can be applied not only to boron cages but also to a wide range of endohedral compounds, one of the most studied (both synthetically^51–53^ and theoretically^54,55^) Sc_3_N@I_h_-C_80_ cluster has been investigated. Detailed charge distribution analysis within Mulliken, Löwdin, Hirshfeld, CM5, QTAIM, and NPA schemes has been performed. For Sc_3_N@I_h_-C_80_ complex, calculated charge values on the Sc_3_N fragment vary from −0.090 e obtained with Mulliken scheme to 0.851 e and 0.969 e for Löwdin and Hirshfeld ones, correspondently. CM5 scheme provides 2.062 e, while NPA and Bader analysis result in 3.426 e and 3.772 e (for more details see ESI). Scheme proposed in current paper predicts the charge on central fragment of 1.502 e based on 1s N orbitals calibration. Thus, with this latter example we show that encapsulated fragment charge estimation scheme described above can be universally used for any endohedral system.

The remarkable feature of this approach is that for fragment X consisting of more than one atom, calibration curve can be constructed using the 1s orbital energy of any of the atoms of the incarcerated molecule. For example, calibration curves constructed using O 1s orbital energies and N 1s orbital energies for NO@B_39_ complex predict 0.212 e and 0.253 e charge on NO species, respectively (in detail, the procedure for constructing the calibration curves and the charges prediction for denoted NO species and all others considered in this paper is presented in supporting information). An agreement between the results obtained from different calibration curves is a good indicator of reliability of the result. However, it should be emphasized that this charge assessment method does not allow determination of charge on the individual atoms of the encapsulated fragment X, but only provides the charge on the whole fragment. On the other hand, taking into account physical model underlying the method described the following limitations must be noted. First, the proposed model does not take into account orbital interactions. Thus, if orbitals of fragment X are involved in strong orbital interactions, a significant error could be observed. Second, because the compensation charge in our method were treated within the framework of point charge model, a small underestimation of the predicted charge could take a place. The reason for this is that charge associated with particular node of cell is not point charge in fact, but only distributed electron density located somewhat closer to the fragment X, which in turn, will certainly affect the accuracy of the electrostatic correction.

## Conclusions

In conclusion, we have developed a new approach for charge assessment of the encapsulated fragment based on the comparison of the low-lying orbital energies of atoms in central fragment in complex of interest with reference systems. The proposed approach demonstrates excellent performance on endohedral borospherenes X@B_39_ with encapsulated metal atoms, halogens or small radicals. Moreover, the workability of proposed approach has been demonstrated on the typical fullerene based Sc_3_N@I_h_-C_80_ endohedral complex. In view of the physical and computational simplicity, the proposed method can be applied to very large systems. In cases when conventional schemes provide essentially different results, the proposed method could be used as a convenient and robust tool to exclude unreliable data from consideration. We are convinced that the proposed approach will find application in research of all kinds of endohedral complexes and related fields of chemical science.

## Electronic supplementary material

Supplementary information
