# Supplementary material for: Reliable charge assessment on encapsulated fragment for endohedral systems
Source: Sci Rep. 2018 Feb 13;8:2882. doi: 10.1038/s41598-018-21240-0 (PMC5811527; doi:10.1038/s41598-018-21240-0)
Supplement: Supplementary file 1 — Supplementary information [file 41598_2018_21240_MOESM1_ESM.pdf]

Supporting Information of

Reliable charge assessment on encapsulated fragment for endohedral systems.

A. J. Stasyuk,*[a] M. Solà*[a] and A.A. Voityuk*[a,b]

Table of contents

		Page
Computational details		S2
Table S1	Charge compensations schemes (CCS1 – CCS5) used for verification of Cl 1s orbital energy dependence on compensation charge distribution method. Units are electrons.	S3
Figure S1	Calibration curves and equations of approximating functions obtained for tested charge compensation schemes (CCS1 – CCS5).	S8
Table S2	Values of Cl 1s orbital energies accessed with various charge compensation schemes (CCS1-CCS5), corrections to electrostatic potentials as well as predicted charges on encapsulated fragment obtained within framework of different CCS for Cl@B39 system. Energies in hartrees and charges in electrons.	S9
Figure S2	Calibration curves and equations of approximating functions constructed for charge prediction on central Cu fragment in Cu@B_{39} complex based on 1s, 2s, and 3s orbital energies.	S10
Figure S3	Graphical representations X@B39 type of systems investigated in present work.	S11
Figure S4	Correlations of the Mulliken, Löwdin, Hirshfeld, CM5, NPA, and QTAIM charges (in electrons) with the results obtained by the proposed method.	S12
Table S3	Slope (α), intercept (β), residual sum of squares (RSS) and R-square (R^2) values for correlations of the Mulliken, Löwdin, Hirshfeld, CM5, NPA, and QTAIM charges with the results obtained by the proposed method.	S13
Table S4	Cartesian coordinates (in Å) for the studied systems.	S14
General description of calibration curves construction for charge assessment on encapsulated fragment for X@YY endohedral system.		S19
Table S5	Charge distribution data and calibration curves for studied systems.	S20
References		S33

Computational details.

Recently we have demonstrated that electronic properties of molecular endohedral borospherenes can be reliably described using DFT.[1] Geometrical structures of the endohedral complexes studied in present work were studied using density functional theory (DFT) with PBE1PBE (hereafter also termed as PBE0) hybrid functional in the ground state. Ahlrichs Def2-TZVP basis sets[2, 3] triple-ξ quality basis set was employed in all cases. All calculations were carried out using Gaussian 09 (Rev. E.01).[4] The population analysis performed within Mulliken[5, 6], Lowdin[7], Hirshfeld[8, 9] and CM5[10] schemes were carried out using code implemented in Gaussian 09. Topological analyses of the electron distributions were conducted according to the "Quantum Theory of Atoms in Molecules" (QTAIM) proposed by Bader.[11, 12] The QTAIM calculations were performed using the AIMALL suite of programs.[13] Natural Population Analysis (NPA) was carried out with NBO 6.0 program.[14]

Table S1. Charge compensations schemes (CCS) used for verification of Cl 1s orbital energy dependence on compensation charge distribution method. Detailed description of particular CCS scheme provide in main text. Units are electrons.

Point charge	CCS1	CCS2	CCS3	CCS4	CCS5
	Cl^{2-}				
q_1	0.05128	-0.04562	0.05150	0.39999	0.58760
q_2	0.05128	0.15307	-0.15840	0.20329	-0.25994
q_3	0.05128	0.14744	0.21500	-0.02467	-0.13984
q_4	0.05128	-0.12873	0.01397	-0.21854	0.09584
q_5	0.05128	0.05693	-0.10389	-0.45001	-0.25040
q_6	0.05128	-0.03604	0.09483	0.43626	0.80456
q_7	0.05128	-0.03602	-0.01656	0.57442	0.60335
q_8	0.05128	0.00714	0.26886	0.28522	0.12787
q_9	0.05128	0.08091	0.09396	-0.34974	-0.16209
q_{10}	0.05128	-0.05192	-0.52281	0.44656	0.65451
q_{11}	0.05128	0.08773	0.40904	-0.14877	-0.15680
q_{12}	0.05128	0.08010	-0.09118	-0.12561	-0.08282
q_{13}	0.05128	0.07533	-0.20293	0.03466	0.11105
q_{14}	0.05128	0.18411	0.21150	0.05128	-0.12070
q_{15}	0.05128	-0.04991	-0.03019	0.56298	0.90852
q_{16}	0.05128	-0.10511	0.36177	0.05110	0.41788
q_{17}	0.05128	0.15151	-0.13516	-0.07971	-0.42133
q_{18}	0.05128	-0.09485	0.34931	0.39922	0.66112
q_{19}	0.05128	0.06707	-0.10362	-0.31363	-0.55948
q_{20}	0.05128	0.17995	0.28964	0.01125	-0.11852
q_{21}	0.05128	0.13477	0.12985	-0.05210	-0.17908
q_{22}	0.05128	0.11394	0.10215	0.13658	-0.12492
q_{23}	0.05128	-0.03320	0.14342	0.05511	0.14868
q_{24}	0.05128	0.11163	-0.04345	-0.40100	-0.47046
q_{25}	0.05128	0.13210	-0.05152	-0.11750	-0.35273
q_{26}	0.05128	0.15541	0.01344	-0.03770	-0.43480
q_{27}	0.05128	0.12277	0.51709	0.14336	-0.10286
q_{28}	0.05128	0.19329	0.20768	-0.00533	-0.51697
q_{29}	0.05128	0.16975	0.25811	0.01566	-0.31878
q_{30}	0.05128	0.06085	-0.19662	0.56713	0.34339
q_{31}	0.05128	-0.02365	-0.44379	0.61305	0.72730
q_{32}	0.05128	-0.04661	0.12142	0.39394	0.72918
q_{33}	0.05128	0.13196	0.06513	-0.03567	-0.30795
q_{34}	0.05128	0.07166	0.06034	-0.16396	0.09423
q_{35}	0.05128	-0.11967	0.12839	-0.19129	0.32029
q_{36}	0.05128	0.05424	-0.22468	0.00716	-0.06257
q_{37}	0.05128	-0.09248	0.01488	0.22938	0.32371

q_{38}	0.05128	0.09819	-0.01584	-0.48744	-0.19846
q_{39}	0.05128	0.04190	0.23497	-0.41478	-0.31753
	Cl^{1-}				
q_{1}	0.02564	-0.02281	0.02575	0.19999	-0.02571
q_{2}	0.02564	0.07653	-0.07920	0.10164	0.01691
q_{3}	0.02564	0.07372	0.10750	-0.01233	0.04183
q_{4}	0.02564	-0.06436	0.00698	-0.10927	0.01936
q_{5}	0.02564	0.02846	-0.05194	-0.22500	0.04913
q_{6}	0.02564	-0.01802	0.04741	0.21813	-0.02129
q_{7}	0.02564	-0.01801	-0.00828	0.28721	-0.01712
q_{8}	0.02564	0.00357	0.13443	0.14261	0.00325
q_{9}	0.02564	0.04045	0.04698	-0.17487	0.06531
q_{10}	0.02564	-0.02596	-0.26140	0.22328	-0.02330
q_{11}	0.02564	0.04386	0.20452	-0.07438	0.05486
q_{12}	0.02564	0.04005	-0.04559	-0.06280	0.06460
q_{13}	0.02564	0.03766	-0.10146	0.01733	0.04599
q_{14}	0.02564	0.09205	0.10575	0.02564	0.02424
q_{15}	0.02564	-0.02495	-0.01509	0.28149	-0.00071
q_{16}	0.02564	-0.05255	0.18088	0.02555	0.02385
q_{17}	0.02564	0.07575	-0.06758	-0.03985	0.04134
q_{18}	0.02564	-0.04742	0.17465	0.19961	-0.00638
q_{19}	0.02564	0.03353	-0.05181	-0.15681	0.06348
q_{20}	0.02564	0.08997	0.14482	0.00562	0.02440
q_{21}	0.02564	0.06738	0.06492	-0.02605	0.03970
q_{22}	0.02564	0.05697	0.05107	0.06829	0.04306
q_{23}	0.02564	-0.01660	0.07171	0.02755	0.00841
q_{24}	0.02564	0.05581	-0.02172	-0.20050	0.06090
q_{25}	0.02564	0.06605	-0.02576	-0.05875	0.03135
q_{26}	0.02564	0.07770	0.00672	-0.01885	0.03621
q_{27}	0.02564	0.06138	0.25854	0.07168	0.02972
q_{28}	0.02564	0.09664	0.10384	-0.00266	0.03351
q_{29}	0.02564	0.08487	0.12905	0.00783	0.02618
q_{30}	0.02564	0.03042	-0.09831	0.28356	-0.02369
q_{31}	0.02564	-0.01182	-0.22189	0.30652	-0.00504
q_{32}	0.02564	-0.02330	0.06071	0.19697	-0.02297
q_{33}	0.02564	0.06598	0.03256	-0.01783	0.04808
q_{34}	0.02564	0.03583	0.03017	-0.08198	0.06231
q_{35}	0.02564	-0.05983	0.06419	-0.09564	0.02918
q_{36}	0.02564	0.02712	-0.11234	0.00358	0.03823
q_{37}	0.02564	-0.04624	0.00744	0.11469	0.00511
q_{38}	0.02564	0.04909	-0.00792	-0.24372	0.05097
q_{39}	0.02564	0.02095	0.11748	-0.20739	0.06463
	Cl^{0}				

q_1	n/a	0.02241	-0.01406	-0.12166	0.22284
q_2	n/a	-0.02268	0.07974	-0.06183	-0.15970
q_3	n/a	-0.02184	-0.05870	0.01165	-0.05419
q_4	n/a	0.06324	-0.00381	0.10322	0.13034
q_5	n/a	-0.00843	0.05230	0.21256	-0.17984
q_6	n/a	0.01770	-0.02589	-0.13269	0.37007
q_7	n/a	0.01769	0.00833	-0.17471	0.30072
q_8	n/a	-0.00105	-0.07341	-0.08675	0.04291
q_9	n/a	-0.01199	-0.02565	0.16519	-0.14591
q_{10}	n/a	0.02550	0.26321	-0.13582	0.26882
q_{11}	n/a	-0.01300	-0.11169	0.07026	-0.07407
q_{12}	n/a	-0.01186	0.04590	0.05933	-0.06338
q_{13}	n/a	-0.01116	0.10216	-0.01054	0.05454
q_{14}	n/a	-0.02728	-0.05775	-0.01559	-0.09940
q_{15}	n/a	0.02452	0.01519	-0.17123	0.22930
q_{16}	n/a	0.05163	-0.09878	-0.01554	0.17128
q_{17}	n/a	-0.02245	0.06804	0.03765	-0.19165
q_{18}	n/a	0.04659	-0.09538	-0.12142	0.21863
q_{19}	n/a	-0.00993	0.05217	0.14814	-0.27054
q_{20}	n/a	-0.02666	-0.07908	-0.00342	-0.08920
q_{21}	n/a	-0.01997	-0.03545	0.02461	-0.06623
q_{22}	n/a	-0.01688	-0.02789	-0.04154	-0.07513
q_{23}	n/a	0.01631	-0.03916	-0.01676	0.05935
q_{24}	n/a	-0.01654	0.02187	0.18940	-0.23557
q_{25}	n/a	-0.01957	0.02593	0.05550	-0.16991
q_{26}	n/a	-0.02302	-0.00367	0.01781	-0.18587
q_{27}	n/a	-0.01819	-0.14119	-0.04360	-0.07406
q_{28}	n/a	-0.02864	-0.05670	0.00251	-0.20717
q_{29}	n/a	-0.02515	-0.07047	-0.00476	-0.13283
q_{30}	n/a	-0.00901	0.09899	-0.17250	0.08140
q_{31}	n/a	0.01162	0.22342	-0.18646	0.27944
q_{32}	n/a	0.02290	-0.03315	-0.11982	0.26538
q_{33}	n/a	-0.01955	-0.01778	0.01684	-0.08966
q_{34}	n/a	-0.01061	-0.01647	0.07744	-0.01237
q_{35}	n/a	0.05879	-0.03505	0.09035	0.21882
q_{36}	n/a	-0.00803	0.12419	-0.00217	-0.07277
q_{37}	n/a	0.04543	-0.00406	-0.06977	0.03810
q_{38}	n/a	-0.01455	0.00797	0.23023	-0.18478
q_{39}	n/a	-0.00620	-0.06415	0.19591	-0.11768
	Cl^{1+}				
q_1	-0.02564	0.02281	-0.02575	-0.20000	-0.09359
q_2	-0.02564	-0.07653	0.07920	-0.10164	-0.03642
q_3	-0.02564	-0.07372	-0.10750	0.01233	0.00794

q_4	-0.02564	0.06436	-0.00698	0.10927	-0.03223
q_5	-0.02564	-0.02846	0.05194	0.22500	0.03194
q_6	-0.02564	0.01802	-0.04741	-0.21813	-0.09084
q_7	-0.02564	0.01801	0.00828	-0.28721	-0.10364
q_8	-0.02564	-0.00357	-0.13443	-0.14261	-0.05233
q_9	-0.02564	-0.04045	-0.04698	0.17487	0.03935
q_{10}	-0.02564	0.02596	0.26140	-0.22328	-0.08111
q_{11}	-0.02564	-0.04386	-0.20452	0.07438	0.01143
q_{12}	-0.02564	-0.04005	0.04559	0.06280	0.01691
q_{13}	-0.02564	-0.03766	0.10146	-0.01733	-0.01770
q_{14}	-0.02564	-0.09205	-0.10575	-0.02564	-0.01542
q_{15}	-0.02564	0.02495	0.01509	-0.28149	-0.09855
q_{16}	-0.02564	0.05255	-0.18088	-0.02555	-0.03373
q_{17}	-0.02564	-0.07575	0.06758	0.03985	0.00377
q_{18}	-0.02564	0.04742	-0.17465	-0.19961	-0.09893
q_{19}	-0.02564	-0.03353	0.05181	0.15681	0.03043
q_{20}	-0.02564	-0.08997	-0.14482	-0.00562	-0.01754
q_{21}	-0.02564	-0.06738	-0.06492	0.02605	0.00471
q_{22}	-0.02564	-0.05697	-0.05107	-0.06829	-0.01896
q_{23}	-0.02564	0.01660	-0.07171	-0.02755	-0.07005
q_{24}	-0.02564	-0.05581	0.02172	0.20050	0.02457
q_{25}	-0.02564	-0.06605	0.02576	0.05875	-0.00850
q_{26}	-0.02564	-0.07770	-0.00672	0.01885	0.00835
q_{27}	-0.02564	-0.06138	-0.25854	-0.07168	-0.01112
q_{28}	-0.02564	-0.09664	-0.10384	0.00266	0.00587
q_{29}	-0.02564	-0.08487	-0.12905	-0.00783	-0.01618
q_{30}	-0.02564	-0.03042	0.09831	-0.28356	-0.09133
q_{31}	-0.02564	0.01182	0.22189	-0.30652	-0.10467
q_{32}	-0.02564	0.02330	-0.06071	-0.19697	-0.08405
q_{33}	-0.02564	-0.06598	-0.03256	0.01783	0.00366
q_{34}	-0.02564	-0.03583	-0.03017	0.08198	0.03067
q_{35}	-0.02564	0.05983	-0.06419	0.09564	-0.03639
q_{36}	-0.02564	-0.02712	0.11234	-0.00358	-0.00753
q_{37}	-0.02564	0.04624	-0.00744	-0.11469	-0.08646
q_{38}	-0.02564	-0.04909	0.00792	0.24372	0.04337
q_{39}	-0.02564	-0.02095	-0.11748	0.20739	0.04439
	Cl^{2+}				
q_1	-0.05128	0.04562	-0.05150	-0.39999	0.33902
q_2	-0.05128	-0.15307	0.15840	-0.20329	-0.22044
q_3	-0.05128	-0.14744	-0.21500	0.02467	-0.07945
q_4	-0.05128	0.12873	-0.01397	0.21854	0.19656
q_5	-0.05128	-0.05693	0.10389	0.45001	-0.28066
q_6	-0.05128	0.03604	-0.09483	-0.43626	0.52121

q_7	-0.05128	0.03602	0.01656	-0.57442	0.35827
q_8	-0.05128	-0.00714	-0.26886	-0.28522	-0.03233
q_9	-0.05128	-0.08091	-0.09396	0.34974	-0.37801
q_{10}	-0.05128	0.05192	0.52281	-0.44656	0.29390
q_{11}	-0.05128	-0.08773	-0.40904	0.14877	-0.10531
q_{12}	-0.05128	-0.08010	0.09118	0.12561	-0.26825
q_{13}	-0.05128	-0.07533	0.20293	-0.03466	0.03227
q_{14}	-0.05128	-0.18411	-0.21150	-0.05128	-0.31749
q_{15}	-0.05128	0.04991	0.03019	-0.56298	0.27189
q_{16}	-0.05128	0.10511	-0.36177	-0.05110	0.31694
q_{17}	-0.05128	-0.15151	0.13516	0.07971	-0.31097
q_{18}	-0.05128	0.09485	-0.34931	-0.39922	0.23307
q_{19}	-0.05128	-0.06707	0.10362	0.31363	-0.38325
q_{20}	-0.05128	-0.17995	-0.28964	-0.01125	-0.13421
q_{21}	-0.05128	-0.13477	-0.12985	0.05210	-0.17795
q_{22}	-0.05128	-0.11394	-0.10215	-0.13658	-0.05687
q_{23}	-0.05128	0.03320	-0.14342	-0.05511	-0.29875
q_{24}	-0.05128	-0.11163	0.04345	0.40100	-0.36550
q_{25}	-0.05128	-0.13210	0.05152	0.11750	-0.32154
q_{26}	-0.05128	-0.15541	-0.01344	0.03770	-0.21742
q_{27}	-0.05128	-0.12277	-0.51709	-0.14336	-0.21230
q_{28}	-0.05128	-0.19329	-0.20768	0.00533	-0.38048
q_{29}	-0.05128	-0.16975	-0.25811	-0.01566	-0.25043
q_{30}	-0.05128	-0.06085	0.19662	-0.56713	0.00594
q_{31}	-0.05128	0.02365	0.44379	-0.61305	0.33145
q_{32}	-0.05128	0.04661	-0.12142	-0.39394	0.36638
q_{33}	-0.05128	-0.13196	-0.06513	0.03567	-0.10962
q_{34}	-0.05128	-0.07166	-0.06034	0.16396	-0.00949
q_{35}	-0.05128	0.11967	-0.12839	0.19129	0.29467
q_{36}	-0.05128	-0.05424	0.22468	-0.00716	-0.12032
q_{37}	-0.05128	0.09248	-0.01488	-0.22938	-0.03576
q_{38}	-0.05128	-0.09819	0.01584	0.48744	-0.31500
q_{39}	-0.05128	-0.04190	-0.23497	0.41478	-0.17971

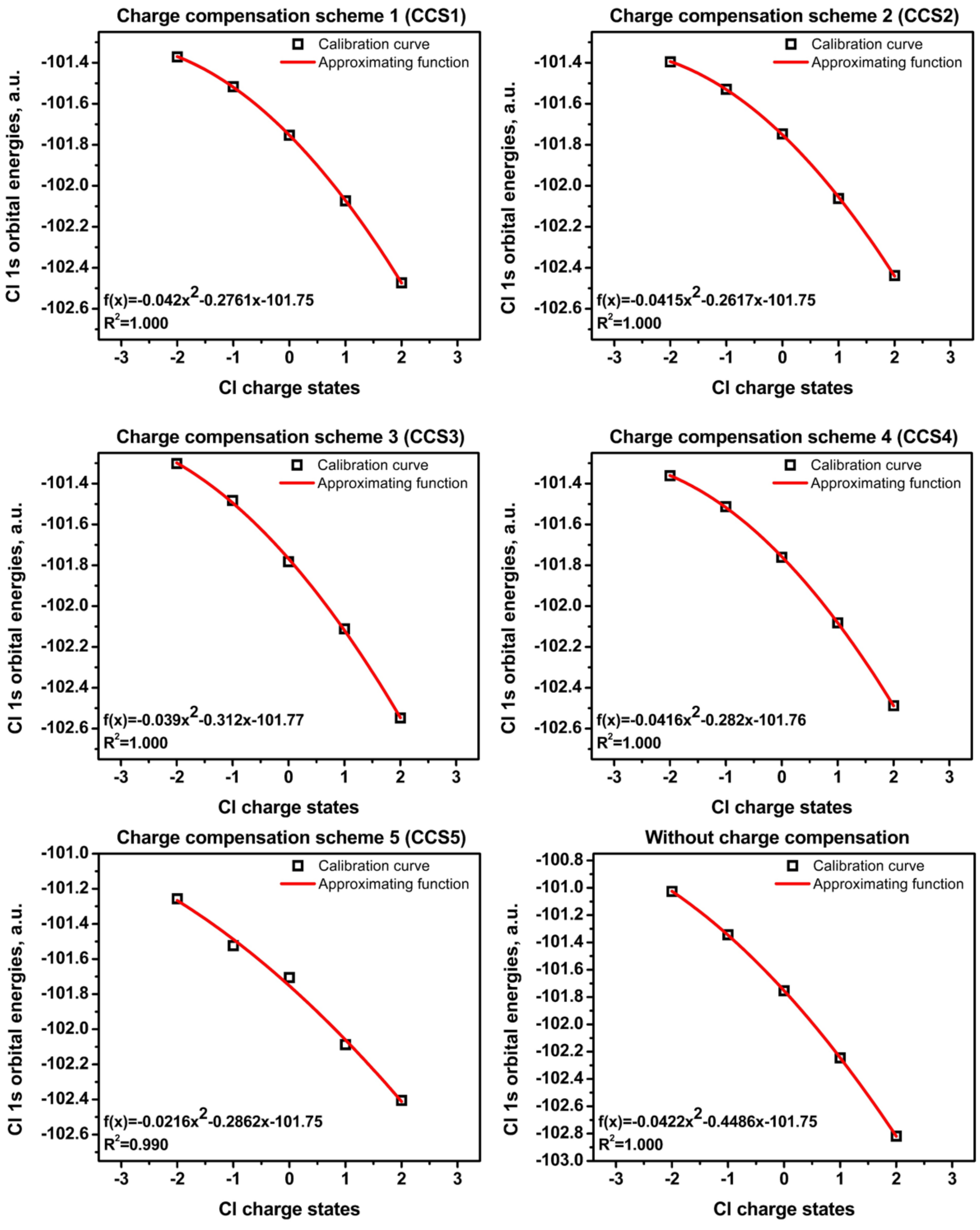

Figure S1. Calibration curves and equations of approximating functions obtained for tested charge compensation schemes (CCS1 – CCS5).

Table S2. Values of Cl 1s orbital energies (ε_{1s}^{Cl}) accessed without (w/o CCS) and with various charge compensation schemes (CCS1-CCS5), corrections to electrostatic potentials (φ_{el}) as well as predicted charge (Q_{Cl}) on encapsulated fragment obtained within framework of different CCS for Cl@B_{39} system.[a] Energies in hartrees and charges in electrons.

Compensation scheme		Charge states					Q_{Cl} predicted
		-2	-1	0	1	2	
w/o CCS	ε_{1s}^{Cl}, a.u.	-101.0261	-101.3441	-101.7538	-102.2460	-102.8180	-0.279
CCS1	ε_{1s}^{Cl}, a.u.	-101.3710	-101.5166	-101.7538	-102.0735	-102.4730	-0.475
	φ_{el}, a.u.	0.3449	0.1725	0.0000	-0.1725	-0.3450	
CCS2	ε_{1s}^{Cl}, a.u.	-101.3956	-101.5291	-101.7472	-102.0620	-102.4377	-0.506
	φ_{el}, a.u.	0.3695	0.1850	-0.0065	-0.1840	-0.3803	
CCS3	ε_{1s}^{Cl}, a.u.	-101.3019	-101.4828	-101.7824	-102.1112	-102.5479	-0.483
	φ_{el}, a.u.	0.2758	0.1387	0.0287	-0.1349	-0.2701	
CCS4	ε_{1s}^{Cl}, a.u.	-101.3618	-101.5135	-101.7605	-102.0818	-102.4879	-0.505
	φ_{el}, a.u.	0.3358	0.1695	0.0068	-0.1643	-0.3301	
CCS5	ε_{1s}^{Cl}, a.u.	-101.2566	-101.5237	-101.7055	-102.0873	-102.4057	-0.440
	φ_{el}, a.u.	0.2306	0.1796	-0.0483	-0.1587	-0.4123	

[a]Cl 1s orbital energy for Cl@B_{39} complex is -101.62826 a.u.

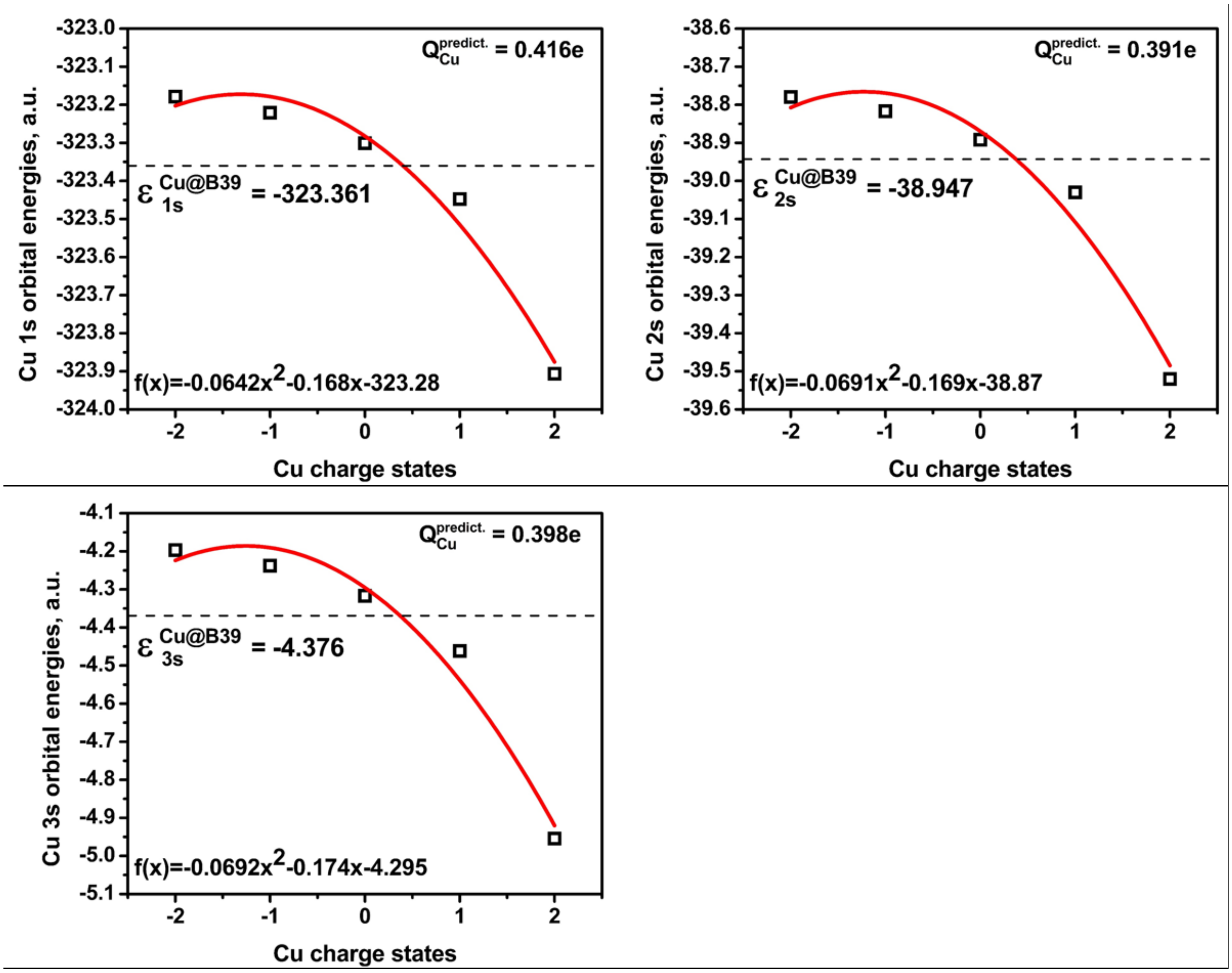

Figure S2. Calibration curves and equations of approximating functions constructed for charge prediction on central Cu fragment in Cu@B_{39} complex based on 1s, 2s, and 3s orbital energies. (Cu 1s, 2s, and 3s orbital energy in complex of interest is -323.361 a.u., -38.947 a.u., and -4.376 a.u. correspondently).

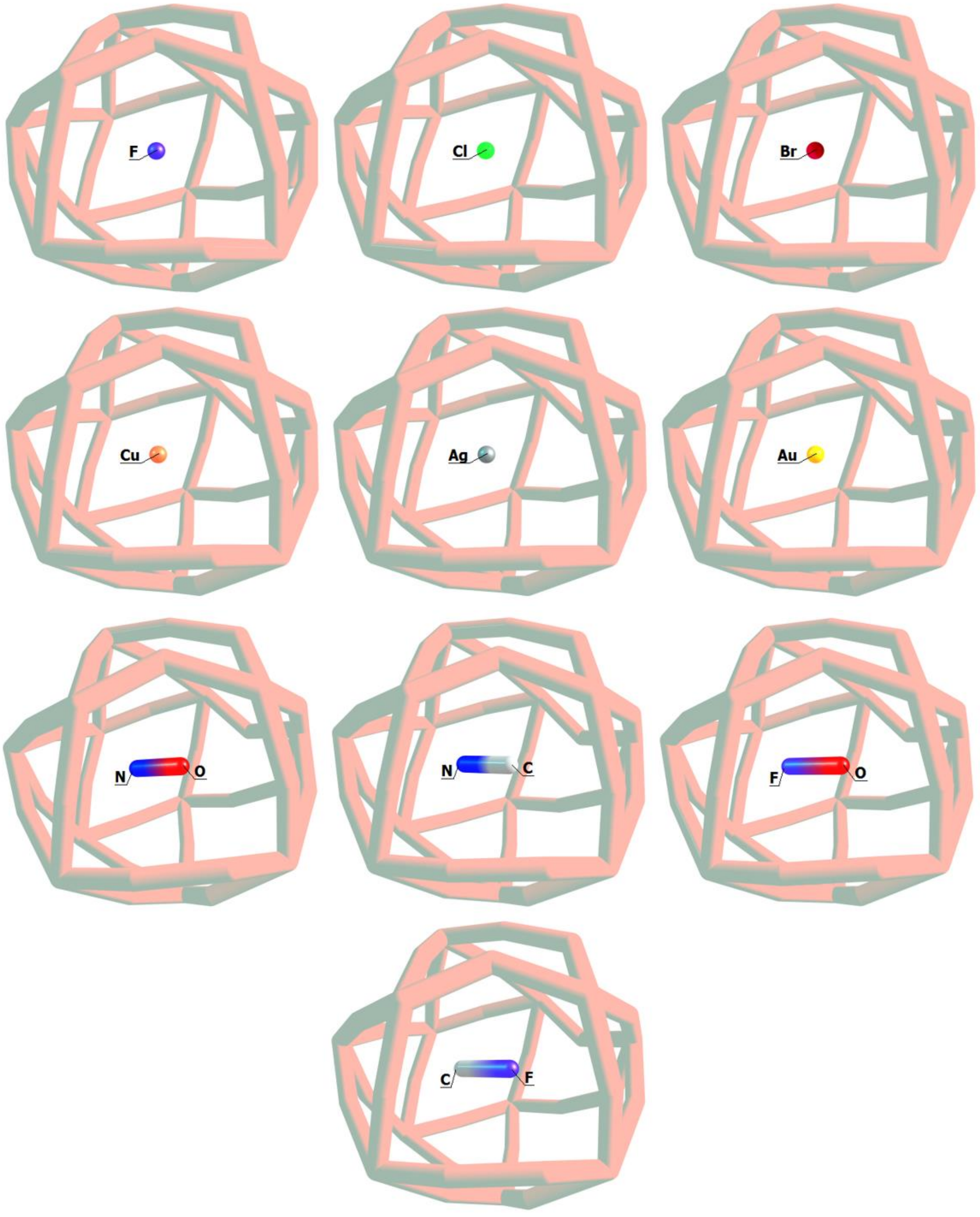

Figure S3. Graphical representations X@B_{39} type of systems investigated in present work.

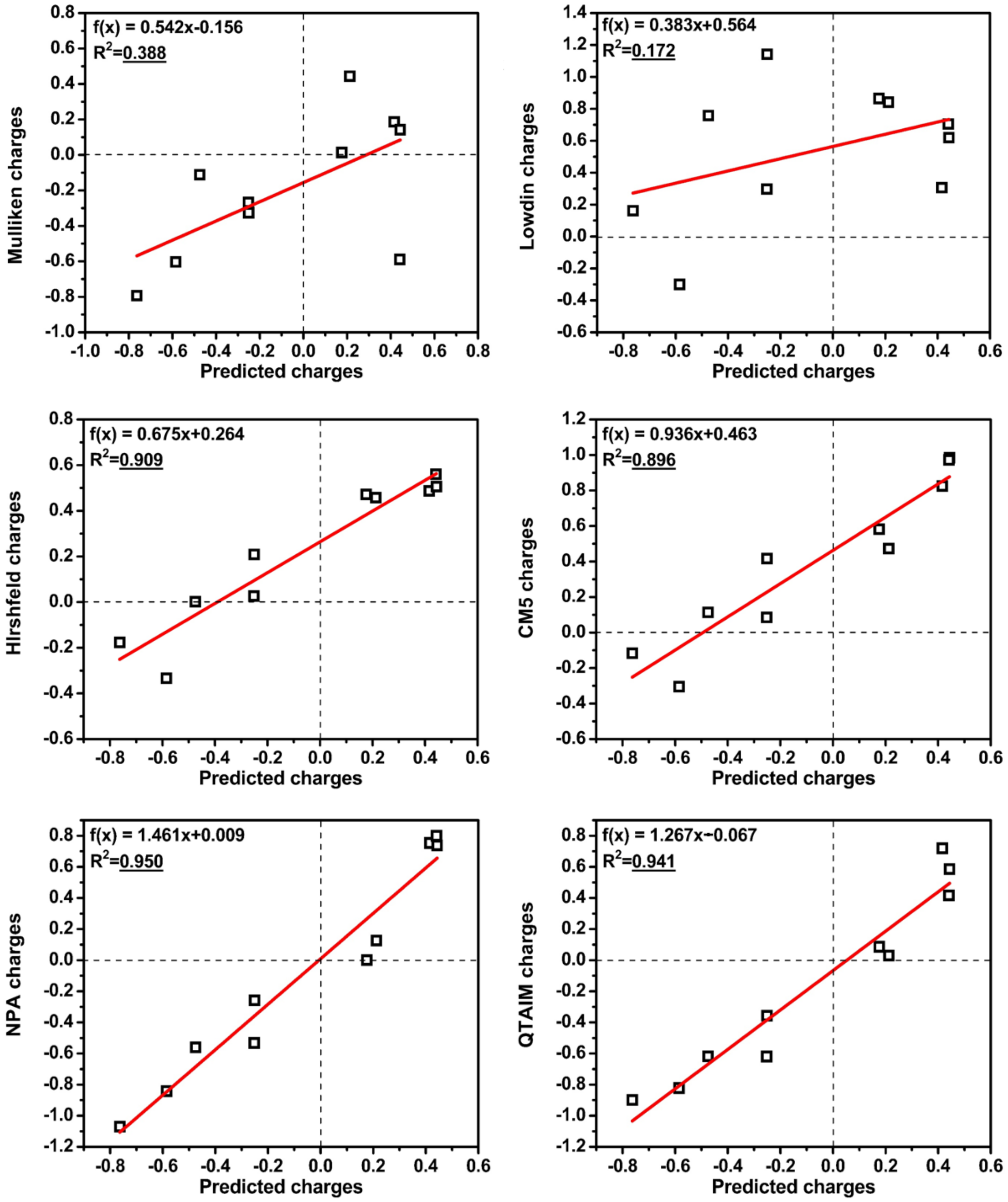

Figure S4. Correlations of the Mulliken, Löwdin, Hirshfeld, CM5, NPA, and QTAIM charges (in electrons) with the results obtained by the proposed method.

Table S3. Slope (α), intercept (β), residual sum of squares (RSS) and R-square (R^2) values for linear correlations of the Mulliken, Löwdin, Hirshfeld, CM5, NPA, and QTAIM charges with the results obtained by the proposed method.

Charge scheme	α	β	RSS	R^2
Mulliken	0.54163	-0.15564	0.86976	0.3875
Löwdin	0.38298	0.56413	1.32758	0.1716
Hirshfeld	0.67453	0.26404	0.08489	0.9094
CM5	0.93576	0.4627	0.19055	0.8960
NPA	1.46096	0.00891	0.21053	0.9500
QTAIM	1.26675	-0.06688	0.19125	0.9402

Cartesian coordinates (in Å) for the studied molecules.

Cl@B_{39}

Atom	X	Y	Z
B	-0.8525729	1.6461958	-2.7756007
B	-2.9197072	0.2090433	0.7349996
B	-1.1690263	2.3266786	-1.3215332
B	1.2528336	-2.0567483	2.2331331
B	2.3543614	-1.5620837	-0.2935620
B	-0.9993609	-1.5614477	-2.7756007
B	1.7057430	2.5539606	-0.8683334
B	-0.2877726	1.8503051	2.8335002
B	0.1756234	2.8199786	-0.2935620
B	-1.7079580	-2.5600143	0.2517388
B	-2.1823839	-1.5042932	1.3745563
B	0.1442372	-1.0279238	3.0668471
B	1.2788167	-2.6330623	0.7349996
B	0.5521702	0.8748793	-3.0344143
B	1.3589233	-2.7541971	-0.8683334
B	-1.4585249	-1.1743710	2.8335002
B	-2.0849250	0.9518814	-1.9342788
B	-3.0646663	0.2002364	-0.8683334
B	-2.5299849	-1.2578949	-0.2935620
B	-1.0337529	0.0407537	-3.0344143
B	-1.4304496	-2.1757458	-1.3215332
B	-0.9623267	0.3890488	3.0668471
B	-2.4076131	-0.0566116	2.2331331
B	-0.1763784	-2.7859879	-0.2670527
B	0.2181090	-2.2815387	-1.9342788
B	1.8668160	1.3296573	-1.9342788
B	-0.2115642	2.6421465	1.3745563
B	-2.3245470	1.5457422	-0.2670527
B	0.4815826	-0.9156331	-3.0344143
B	-1.3630584	2.7591422	0.2517388
B	3.0710164	-0.1991279	0.2517388
B	1.8519339	-0.0847480	-2.7756007
B	2.5994759	-0.1509327	-1.3215332
B	0.8180895	0.6388750	3.0668471
B	1.1547794	2.1133599	2.2331331
B	1.6408905	2.4240189	0.7349996
B	1.7462975	-0.6759341	2.8335002
B	2.5009255	1.2402456	-0.2670527
B	2.3939481	-1.1378532	1.3745563
Cl	0.0000000	0.0000000	0.0000000

(B_{39}+Cl) VdW

Atom	X	Y	Z
B	-0.8525729	1.6461958	-2.7756007
B	-2.9197072	0.2090433	0.7349996
B	-1.1690263	2.3266786	-1.3215332
B	1.2528336	-2.0567483	2.2331331
B	2.3543614	-1.5620837	-0.2935620
B	-0.9993609	-1.5614477	-2.7756007
B	1.7057430	2.5539606	-0.8683334
B	-0.2877726	1.8503051	2.8335002
B	0.1756234	2.8199786	-0.2935620
B	-1.7079580	-2.5600143	0.2517388
B	-2.1823839	-1.5042932	1.3745563
B	0.1442372	-1.0279238	3.0668471
B	1.2788167	-2.6330623	0.7349996
B	0.5521702	0.8748793	-3.0344143
B	1.3589233	-2.7541971	-0.8683334
B	-1.4585249	-1.1743710	2.8335002
B	-2.0849250	0.9518814	-1.9342788
B	-3.0646663	0.2002364	-0.8683334
B	-2.5299849	-1.2578949	-0.2935620
B	-1.0337529	0.0407537	-3.0344143
B	-1.4304496	-2.1757458	-1.3215332
B	-0.9623267	0.3890488	3.0668471
B	-2.4076131	-0.0566116	2.2331331
B	-0.1763784	-2.7859879	-0.2670527
B	0.2181090	-2.2815387	-1.9342788
B	1.8668160	1.3296573	-1.9342788
B	-0.2115642	2.6421465	1.3745563
B	-2.3245470	1.5457422	-0.2670527
B	0.4815826	-0.9156331	-3.0344143
B	-1.3630584	2.7591422	0.2517388
B	3.0710164	-0.1991279	0.2517388
B	1.8519339	-0.0847480	-2.7756007
B	2.5994759	-0.1509327	-1.3215332
B	0.8180895	0.6388750	3.0668471
B	1.1547794	2.1133599	2.2331331
B	1.6408905	2.4240189	0.7349996
B	1.7462975	-0.6759341	2.8335002
B	2.5009255	1.2402456	-0.2670527
B	2.3939481	-1.1378532	1.3745563
Cl	0.0000000	-3.0000000	0.0000000

$X@B_{39}^{+}$ (X = Cu, Ag, Au, F, Br)

Atom	X	Y	Z
B	-0.8525729	1.6461958	-2.7756007
B	-2.9197072	0.2090433	0.7349996
B	-1.1690263	2.3266786	-1.3215332
B	1.2528336	-2.0567483	2.2331331
B	2.3543614	-1.5620837	-0.2935620
B	-0.9993609	-1.5614477	-2.7756007
B	1.7057430	2.5539606	-0.8683334
B	-0.2877726	1.8503051	2.8335002
B	0.1756234	2.8199786	-0.2935620
B	-1.7079580	-2.5600143	0.2517388
B	-2.1823839	-1.5042932	1.3745563
B	0.1442372	-1.0279238	3.0668471
B	1.2788167	-2.6330623	0.7349996
B	0.5521702	0.8748793	-3.0344143
B	1.3589233	-2.7541971	-0.8683334
B	-1.4585249	-1.1743710	2.8335002
B	-2.0849250	0.9518814	-1.9342788
B	-3.0646663	0.2002364	-0.8683334
B	-2.5299849	-1.2578949	-0.2935620
B	-1.0337529	0.0407537	-3.0344143
B	-1.4304496	-2.1757458	-1.3215332
B	-0.9623267	0.3890488	3.0668471
B	-2.4076131	-0.0566116	2.2331331
B	-0.1763784	-2.7859879	-0.2670527
B	0.2181090	-2.2815387	-1.9342788
B	1.8668160	1.3296573	-1.9342788
B	-0.2115642	2.6421465	1.3745563
B	-2.3245470	1.5457422	-0.2670527
B	0.4815826	-0.9156331	-3.0344143
B	-1.3630584	2.7591422	0.2517388
B	3.0710164	-0.1991279	0.2517388
B	1.8519339	-0.0847480	-2.7756007
B	2.5994759	-0.1509327	-1.3215332
B	0.8180895	0.6388750	3.0668471
B	1.1547794	2.1133599	2.2331331
B	1.6408905	2.4240189	0.7349996
B	1.7462975	-0.6759341	2.8335002
B	2.5009255	1.2402456	-0.2670527
B	2.3939481	-1.1378532	1.3745563
X	0.0000000	0.0000000	0.0000000

$NO@B_{39}$

Atom	X	Y	Z
B	-0.8525729	1.6461958	-2.7756007
B	-2.9197072	0.2090433	0.7349996
B	-1.1690263	2.3266786	-1.3215332
B	1.2528336	-2.0567483	2.2331331
B	2.3543614	-1.5620837	-0.2935620
B	-0.9993609	-1.5614477	-2.7756007
B	1.7057430	2.5539606	-0.8683334
B	-0.2877726	1.8503051	2.8335002
B	0.1756234	2.8199786	-0.2935620
B	-1.7079580	-2.5600143	0.2517388
B	-2.1823839	-1.5042932	1.3745563
B	0.1442372	-1.0279238	3.0668471
B	1.2788167	-2.6330623	0.7349996
B	0.5521702	0.8748793	-3.0344143
B	1.3589233	-2.7541971	-0.8683334
B	-1.4585249	-1.1743710	2.8335002
B	-2.0849250	0.9518814	-1.9342788
B	-3.0646663	0.2002364	-0.8683334
B	-2.5299849	-1.2578949	-0.2935620
B	-1.0337529	0.0407537	-3.0344143
B	-1.4304496	-2.1757458	-1.3215332
B	-0.9623267	0.3890488	3.0668471
B	-2.4076131	-0.0566116	2.2331331
B	-0.1763784	-2.7859879	-0.2670527
B	0.2181090	-2.2815387	-1.9342788
B	1.8668160	1.3296573	-1.9342788
B	-0.2115642	2.6421465	1.3745563
B	-2.3245470	1.5457422	-0.2670527
B	0.4815826	-0.9156331	-3.0344143
B	-1.3630584	2.7591422	0.2517388
B	3.0710164	-0.1991279	0.2517388
B	1.8519339	-0.0847480	-2.7756007
B	2.5994759	-0.1509327	-1.3215332
B	0.8180895	0.6388750	3.0668471
B	1.1547794	2.1133599	2.2331331
B	1.6408905	2.4240189	0.7349996
B	1.7462975	-0.6759341	2.8335002
B	2.5009255	1.2402456	-0.2670527
B	2.3939481	-1.1378532	1.3745563
N	0.0000000	0.0000000	0.5698790
O	0.0000000	0.0000000	-0.5698790

CN@B_{39}

Atom	X	Y	Z
B	-0.8525729	1.6461958	-2.7756007
B	-2.9197072	0.2090433	0.7349996
B	-1.1690263	2.3266786	-1.3215332
B	1.2528336	-2.0567483	2.2331331
B	2.3543614	-1.5620837	-0.2935620
B	-0.9993609	-1.5614477	-2.7756007
B	1.7057430	2.5539606	-0.8683334
B	-0.2877726	1.8503051	2.8335002
B	0.1756234	2.8199786	-0.2935620
B	-1.7079580	-2.5600143	0.2517388
B	-2.1823839	-1.5042932	1.3745563
B	0.1442372	-1.0279238	3.0668471
B	1.2788167	-2.6330623	0.7349996
B	0.5521702	0.8748793	-3.0344143
B	1.3589233	-2.7541971	-0.8683334
B	-1.4585249	-1.1743710	2.8335002
B	-2.0849250	0.9518814	-1.9342788
B	-3.0646663	0.2002364	-0.8683334
B	-2.5299849	-1.2578949	-0.2935620
B	-1.0337529	0.0407537	-3.0344143
B	-1.4304496	-2.1757458	-1.3215332
B	-0.9623267	0.3890488	3.0668471
B	-2.4076131	-0.0566116	2.2331331
B	-0.1763784	-2.7859879	-0.2670527
B	0.2181090	-2.2815387	-1.9342788
B	1.8668160	1.3296573	-1.9342788
B	-0.2115642	2.6421465	1.3745563
B	-2.3245470	1.5457422	-0.2670527
B	0.4815826	-0.9156331	-3.0344143
B	-1.3630584	2.7591422	0.2517388
B	3.0710164	-0.1991279	0.2517388
B	1.8519339	-0.0847480	-2.7756007
B	2.5994759	-0.1509327	-1.3215332
B	0.8180895	0.6388750	3.0668471
B	1.1547794	2.1133599	2.2331331
B	1.6408905	2.4240189	0.7349996
B	1.7462975	-0.6759341	2.8335002
B	2.5009255	1.2402456	-0.2670527
B	2.3939481	-1.1378532	1.3745563
C	0.0000000	0.0000000	0.5811170
N	0.0000000	0.0000000	-0.5811170

FO@B_{39}

Atom	X	Y	Z
B	-0.8525729	1.6461958	-2.7756007
B	-2.9197072	0.2090433	0.7349996
B	-1.1690263	2.3266786	-1.3215332
B	1.2528336	-2.0567483	2.2331331
B	2.3543614	-1.5620837	-0.2935620
B	-0.9993609	-1.5614477	-2.7756007
B	1.7057430	2.5539606	-0.8683334
B	-0.2877726	1.8503051	2.8335002
B	0.1756234	2.8199786	-0.2935620
B	-1.7079580	-2.5600143	0.2517388
B	-2.1823839	-1.5042932	1.3745563
B	0.1442372	-1.0279238	3.0668471
B	1.2788167	-2.6330623	0.7349996
B	0.5521702	0.8748793	-3.0344143
B	1.3589233	-2.7541971	-0.8683334
B	-1.4585249	-1.1743710	2.8335002
B	-2.0849250	0.9518814	-1.9342788
B	-3.0646663	0.2002364	-0.8683334
B	-2.5299849	-1.2578949	-0.2935620
B	-1.0337529	0.0407537	-3.0344143
B	-1.4304496	-2.1757458	-1.3215332
B	-0.9623267	0.3890488	3.0668471
B	-2.4076131	-0.0566116	2.2331331
B	-0.1763784	-2.7859879	-0.2670527
B	0.2181090	-2.2815387	-1.9342788
B	1.8668160	1.3296573	-1.9342788
B	-0.2115642	2.6421465	1.3745563
B	-2.3245470	1.5457422	-0.2670527
B	0.4815826	-0.9156331	-3.0344143
B	-1.3630584	2.7591422	0.2517388
B	3.0710164	-0.1991279	0.2517388
B	1.8519339	-0.0847480	-2.7756007
B	2.5994759	-0.1509327	-1.3215332
B	0.8180895	0.6388750	3.0668471
B	1.1547794	2.1133599	2.2331331
B	1.6408905	2.4240189	0.7349996
B	1.7462975	-0.6759341	2.8335002
B	2.5009255	1.2402456	-0.2670527
B	2.3939481	-1.1378532	1.3745563
F	0.0000000	0.0000000	-0.6743775
O	0.0000000	0.0000000	0.6743775

CF@B_{39}

Atom	X	Y	Z
B	-0.8525729	1.6461958	-2.7756007
B	-2.9197072	0.2090433	0.7349996
B	-1.1690263	2.3266786	-1.3215332
B	1.2528336	-2.0567483	2.2331331
B	2.3543614	-1.5620837	-0.2935620
B	-0.9993609	-1.5614477	-2.7756007
B	1.7057430	2.5539606	-0.8683334
B	-0.2877726	1.8503051	2.8335002
B	0.1756234	2.8199786	-0.2935620
B	-1.7079580	-2.5600143	0.2517388
B	-2.1823839	-1.5042932	1.3745563
B	0.1442372	-1.0279238	3.0668471
B	1.2788167	-2.6330623	0.7349996
B	0.5521702	0.8748793	-3.0344143
B	1.3589233	-2.7541971	-0.8683334
B	-1.4585249	-1.1743710	2.8335002
B	-2.0849250	0.9518814	-1.9342788
B	-3.0646663	0.2002364	-0.8683334
B	-2.5299849	-1.2578949	-0.2935620
B	-1.0337529	0.0407537	-3.0344143
B	-1.4304496	-2.1757458	-1.3215332
B	-0.9623267	0.3890488	3.0668471
B	-2.4076131	-0.0566116	2.2331331
B	-0.1763784	-2.7859879	-0.2670527
B	0.2181090	-2.2815387	-1.9342788
B	1.8668160	1.3296573	-1.9342788
B	-0.2115642	2.6421465	1.3745563
B	-2.3245470	1.5457422	-0.2670527
B	0.4815826	-0.9156331	-3.0344143
B	-1.3630584	2.7591422	0.2517388
B	3.0710164	-0.1991279	0.2517388
B	1.8519339	-0.0847480	-2.7756007
B	2.5994759	-0.1509327	-1.3215332
B	0.8180895	0.6388750	3.0668471
B	1.1547794	2.1133599	2.2331331
B	1.6408905	2.4240189	0.7349996
B	1.7462975	-0.6759341	2.8335002
B	2.5009255	1.2402456	-0.2670527
B	2.3939481	-1.1378532	1.3745563
C	0.0000000	0.0000000	0.6341220
F	0.0000000	0.0000000	-0.6341220

Sc_3N@I_h-C_{80}

Atom	X	Y	Z
C	2.0969437	0.3049768	0.3301716
C	-0.8556314	-1.4574183	0.8355135
C	-0.7048720	1.0165015	-1.5965134
C	0.1854454	-0.0588864	-0.1565573
C	-3.1511421	2.3855960	-0.2307880
C	-3.5857610	1.2805242	-1.0062417
C	-3.0672152	1.1729197	-2.3481687
C	4.0701106	-0.0254233	-1.3119469
C	-2.9799369	2.2907628	1.1869176
C	-2.4053564	0.7393536	2.9755701
C	-3.2258515	1.0592857	1.8601897
C	-3.7390658	-0.0268086	1.0955989
C	-3.6741027	-1.2097291	-0.9055681
C	-3.0688530	-1.3421964	-2.1836253
C	-2.7809651	-0.1274230	-2.8896550
C	-2.2112803	-2.4448714	-2.4182447
C	-3.3385882	-2.1208759	0.1539609
C	-1.6347107	-3.6221916	1.0894725
C	-2.4312531	-3.2092200	-0.0470873
C	-1.8804602	-3.3585724	-1.3575576
C	-2.0478336	-0.6242847	3.2702823
C	-2.5461376	-1.7328926	2.5135159
C	-3.3856440	-1.3898260	1.4023644
C	-1.6950898	-2.9047558	2.3714562
C	0.6440761	2.7548889	-3.0946401
C	0.1649084	1.6554899	-3.8751922
C	-1.6567153	-0.0122449	-3.7672458
C	-1.2546842	1.3762374	-3.8186689
C	-0.2260727	3.4906334	-2.2018242
C	-2.1522638	3.2921474	-0.7480052
C	-1.6197680	3.1772077	-2.0723874
C	-2.1539380	2.1364213	-2.9382857
C	0.8747517	3.7700913	1.3296367
C	0.0188951	4.0374702	0.2221900
C	0.5710388	3.9350967	-1.0863159
C	-1.3642488	3.7460370	0.3689252
C	2.0129806	-0.0143059	3.5353222
C	0.1759922	-1.6790262	3.6004766
C	1.5640734	-1.3754742	3.4915507
C	-0.3294687	-2.8067019	2.8562794
C	3.1668938	0.1049220	2.6796571
C	4.1497468	1.2010457	0.6981333
C	3.4877624	1.3204794	1.9888808
C	2.5550775	2.3918263	2.1715929
C	3.4914920	-2.4364242	-0.0040667

C	3.9609163	-1.3375940	0.7799342
C	3.4117622	-1.1959540	2.0936077
C	4.3646248	-0.1248302	0.1003711
C	0.5381600	-3.4895613	1.9361370
C	1.9235526	-3.1528399	1.7814525
C	2.4206992	-2.1043079	2.6005526
C	2.4957439	-3.3437242	0.4999793
C	-0.2513414	-3.9651280	0.8414437
C	-0.5301694	-3.8123505	-1.5761533
C	0.3237184	-4.0796165	-0.4744508
C	1.7095703	-3.8005934	-0.6120346
C	3.6957714	2.0695012	-0.3745106
C	2.7409987	3.1351193	-0.1907745
C	2.2141440	3.3148294	1.1137269
C	1.9192370	3.4946433	-1.2981597
C	0.3738066	3.1262771	2.5089263
C	-0.9986374	2.7581959	2.6209109
C	-1.8716780	3.1227555	1.5549540
C	-1.3105969	1.5799483	3.3595553
C	1.4063256	2.2834071	3.0338548
C	-0.7218057	-0.6084096	3.8432490
C	1.0987100	1.0652075	3.6980641
C	-0.2755195	0.7499932	3.9036674
C	3.6881855	1.3225724	-1.6025747
C	2.3894448	0.5715678	-3.5169921
C	2.8246503	1.6445961	-2.7012547
C	1.9660211	2.7632338	-2.5317233
C	3.3139975	-2.3402976	-1.4307539
C	2.7503371	-0.7876862	-3.2247388
C	3.5582495	-1.1146615	-2.1032017
C	-3.9252500	0.0770300	-0.3225307
C	-0.0311178	-3.1797283	-2.7590616
C	1.6560573	-1.6336147	-3.6083165
C	1.3405657	-2.8079269	-2.8653664
C	2.2148086	-3.1781069	-1.8027518
Sc	-1.0685179	-2.3364308	-3.2815379
Sc	1.0675951	0.5671618	-4.0841091
Sc	-0.7484115	-1.1175925	-3.9368607
N	0.6201275	-0.8012561	-4.1403934

General description of calibration curves construction for charge assessment on encapsulated fragment for X@YY endohedral system.

1. At the initial stage, 1s orbital energies for encapsulated fragment X taken in various charge states has been obtained.
2. At the next stage calibration curve of 1s orbital energy on charge state for specie of interest has been constructed.
3. Charge prediction is performed on the basis of the calibration curve obtained above and the 1s orbital energy value for encapsulated fragment X in X@YY system.

In the case when encapsulated fragment represented by a group of atoms calibration curve as well as charge prediction can be performed for any of the atoms constituting the fragment X.

Cl+B39 (VdW) [0,1]		Mulliken Scheme	Lowdin Scheme	Hirshfeld Scheme	CM5 Scheme	NPA Scheme	Bader Scheme
Atom Number	Element	Charge density	Charge density	Charge density	Charge density	Charge density	Charge density
1	B	0.1732	0.0198	-0.0696	-0.0696	-0.1962	6.93E-02
2	B	0.0095	0.0017	0.0185	0.0185	0.0339	1.23E-01
3	B	-0.0488	-0.0309	0.0229	0.0229	0.0455	-5.73E-02
4	B	0.1000	0.0544	0.0062	0.0061	0.0318	4.76E-03
5	B	-0.1612	0.0044	0.0537	0.0537	0.1528	2.27E-02
6	B	0.2034	0.0187	-0.0707	-0.0707	-0.2103	4.55E-02
7	B	0.2119	0.0198	-0.0647	-0.0647	-0.2076	3.31E-02
8	B	0.2114	0.0434	0.0015	0.0010	-0.0506	5.69E-02
9	B	-0.2075	-0.0062	0.0465	0.0465	0.1178	-1.64E-02
10	B	0.0958	0.0150	-0.0642	-0.0642	-0.2009	2.18E-01
11	B	-0.1235	-0.0058	0.0427	0.0427	0.0863	-1.73E-01
12	B	-0.1158	-0.0415	0.0639	0.0617	0.1323	3.95E-02
13	B	0.0690	-0.0135	0.0074	0.0074	-0.0241	5.66E-02
14	B	-0.0317	-0.0014	0.0380	0.0380	0.1070	-4.51E-02
15	B	0.2076	0.0256	-0.0630	-0.0630	-0.2011	2.01E-02
16	B	0.2185	0.0359	-0.0047	-0.0052	-0.0855	-6.48E-02
17	B	-0.0918	-0.0113	0.0379	0.0379	0.1044	4.77E-02
18	B	0.1784	0.0214	-0.0669	-0.0669	-0.2240	-8.79E-02
19	B	-0.1421	-0.0046	0.0471	0.0471	0.1285	3.77E-02
20	B	-0.0280	-0.0043	0.0372	0.0372	0.1097	-7.76E-02
21	B	-0.0481	-0.0247	0.0276	0.0276	0.0684	-3.17E-02
22	B	-0.0271	-0.0069	0.0764	0.0742	0.1845	8.52E-02
23	B	0.0551	0.0599	0.0053	0.0052	0.0345	5.87E-02
24	B	-0.2079	-0.0147	0.0442	0.0442	0.1290	-1.57E-02
25	B	-0.0684	-0.0036	0.0404	0.0404	0.1230	3.87E-02
26	B	-0.0909	-0.0052	0.0372	0.0372	0.1016	1.17E-02
27	B	-0.0217	0.0276	0.0503	0.0503	0.1184	-1.44E-01
28	B	-0.1593	0.0019	0.0490	0.0490	0.1477	1.24E-03
29	B	-0.0489	-0.0031	0.0371	0.0371	0.1051	-4.42E-02
30	B	0.1305	0.0155	-0.0616	-0.0616	-0.1862	2.07E-01
31	B	0.1451	0.0156	-0.0678	-0.0678	-0.2367	1.86E-01
32	B	0.2000	0.0206	-0.0678	-0.0678	-0.1985	3.18E-02
33	B	-0.0073	-0.0195	0.0297	0.0297	0.0704	-2.06E-02
34	B	-0.1594	-0.0394	0.0637	0.0615	0.1390	9.79E-03
35	B	0.1026	0.0413	-0.0014	-0.0014	-0.0065	-2.78E-02
36	B	0.0317	-0.0113	0.0081	0.0081	-0.0022	7.22E-02
37	B	0.2404	0.0417	-0.0033	-0.0040	-0.0677	6.65E-02
38	B	-0.2416	-0.0090	0.0528	0.0528	0.1665	-2.02E-02
39	B	-0.1435	0.0117	0.0543	0.0543	0.1401	-1.38E-01
40	Cl	-0.4098	-0.2392	-0.3935	-0.3851	-0.4803	-5.47E-01
	F1(B39)	0.4098	0.2392	0.3935	0.3851	0.4803	0.580
	F2(Cl)	-0.4098	-0.2392	-0.3935	-0.3851	-0.4803	-0.547
	Total	0.0000	0.0000	0.0000	0.0000	0.0000	0.033

F@B39 [0,1]		Mulliken Scheme	Lowdin Scheme	Hirshfeld Scheme	CM5 Scheme	NPA Scheme	Bader Scheme
Atom Number	Element	Charge density	Charge density	Charge density	Charge density	Charge density	Charge density
1	B	0.1870	0.0150	-0.0751	-0.0755	-0.2092	0.0497
2	B	-0.0539	-0.0140	0.0017	0.0009	-0.0373	0.1462
3	B	-0.0060	-0.0289	0.0250	0.0240	0.0734	-0.0011
4	B	0.2197	0.0461	0.0007	0.0003	0.0777	-0.0122
5	B	-0.1540	-0.0016	0.0542	0.0529	0.1732	0.0646
6	B	0.1870	0.0150	-0.0750	-0.0754	-0.2105	0.0467
7	B	0.2249	0.0305	-0.0550	-0.0555	-0.2012	-0.0481
8	B	0.2080	0.0278	-0.0285	-0.0288	-0.1146	-0.0338
9	B	-0.1540	-0.0016	0.0541	0.0529	0.1832	0.0646
10	B	0.1522	0.0112	-0.0721	-0.0728	-0.2501	0.2267
11	B	-0.1558	0.0171	0.0638	0.0629	0.1990	-0.1515
12	B	-0.1301	0.0052	0.0553	0.0548	0.0882	0.0146
13	B	-0.0539	-0.0140	0.0018	0.0010	-0.0379	0.1462
14	B	-0.0405	-0.0029	0.0375	0.0370	0.1189	-0.0416
15	B	0.2249	0.0305	-0.0550	-0.0555	-0.1930	-0.0509
16	B	0.2080	0.0278	-0.0286	-0.0289	-0.1151	-0.0357
17	B	-0.0832	0.0009	0.0467	0.0458	0.1375	0.0597
18	B	0.2249	0.0305	-0.0550	-0.0555	-0.1937	-0.0501
19	B	-0.1540	-0.0016	0.0542	0.0529	0.1815	0.0648
20	B	-0.0405	-0.0029	0.0376	0.0371	0.1179	-0.0414
21	B	-0.0060	-0.0289	0.0252	0.0241	0.0713	-0.0010
22	B	-0.1301	0.0052	0.0553	0.0548	0.0914	0.0145
23	B	0.2197	0.0461	0.0007	0.0002	0.0729	-0.0126
24	B	-0.1674	-0.0063	0.0568	0.0555	0.2126	0.0252
25	B	-0.0832	0.0009	0.0468	0.0459	0.1412	0.0596
26	B	-0.0832	0.0009	0.0467	0.0458	0.1394	0.0595
27	B	-0.1558	0.0171	0.0639	0.0630	0.1984	-0.1515
28	B	-0.1674	-0.0063	0.0568	0.0554	0.2063	0.0250
29	B	-0.0405	-0.0029	0.0376	0.0371	0.1183	-0.0416
30	B	0.1522	0.0112	-0.0721	-0.0727	-0.2377	0.2260
31	B	0.1522	0.0112	-0.0720	-0.0727	-0.2357	0.2256
32	B	0.1870	0.0150	-0.0751	-0.0755	-0.2144	0.0468
33	B	-0.0060	-0.0289	0.0251	0.0240	0.0752	-0.0010
34	B	-0.1301	0.0052	0.0553	0.0548	0.0889	0.0146
35	B	0.2197	0.0461	0.0007	0.0003	0.0728	-0.0131
36	B	-0.0539	-0.0140	0.0018	0.0010	-0.0412	0.1463
37	B	0.2080	0.0278	-0.0285	-0.0289	-0.1094	-0.0370
38	B	-0.1674	-0.0063	0.0569	0.0555	0.2117	0.0251
39	B	-0.1558	0.0171	0.0640	0.0631	0.1926	-0.1515
40	F	-0.6031	-0.3004	-0.3336	-0.3051	-0.8424	-0.8221
	F1(B39)	0.6031	0.3004	0.3339	0.3055	0.8424	0.8765
	F2(F)	-0.6031	-0.3004	-0.3336	-0.3051	-0.8424	-0.8221
	Total	0.0000	0.0000	0.0003	0.0003	0.0000	0.0544

F calibration curve constraction:

Charges on F	F 1s orbital energies
-2	-24.21387
-1	-24.42497
0	-24.86812
1	-25.46937
2	-26.23521

Orbital energies in F@B39 complex	
F 1s	-24.59440

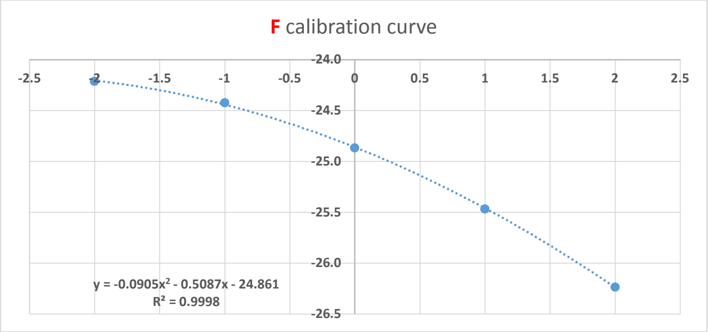

$$Q_{predicted} = -0.585$$

Cl@B39 [0,1]		Mulliken Scheme	Lowdin Scheme	Hirshfeld Scheme	CM5 Scheme	NPA Scheme	Bader Scheme
Atom Number	Element	Charge density	Charge density	Charge density	Charge density	Charge density	Charge density
1	B	0.3405	0.0122	-0.0639	-0.0653	-0.1689	-1.63E-02
2	B	-0.0502	-0.0319	0.0009	-0.0022	-0.0146	1.08E-01
3	B	-0.0605	-0.0627	0.0153	0.0112	0.0603	-1.70E-03
4	B	0.2794	0.0354	-0.0018	-0.0034	0.0835	-1.38E-02
5	B	-0.3442	-0.0528	0.0422	0.0373	0.1659	1.99E-02
6	B	0.3268	0.0092	-0.0672	-0.0686	-0.1735	1.40E-02
7	B	0.3759	0.0105	-0.0634	-0.0655	-0.2058	-5.22E-02
8	B	0.2918	0.0153	-0.0287	-0.0299	-0.0698	-5.58E-02
9	B	-0.3331	-0.0538	0.0372	0.0323	0.1499	6.82E-02
10	B	0.2735	-0.0087	-0.0720	-0.0747	-0.2142	2.01E-01
11	B	-0.2486	-0.0283	0.0330	0.0295	0.1032	-1.48E-01
12	B	-0.2195	-0.0200	0.0354	0.0335	0.0276	2.69E-02
13	B	-0.0230	-0.0375	-0.0003	-0.0035	0.0023	1.15E-01
14	B	-0.1047	-0.0152	0.0239	0.0219	0.0572	-5.86E-03
15	B	0.3285	-0.0014	-0.0768	-0.0789	-0.2152	-2.65E-02
16	B	0.3185	0.0237	-0.0162	-0.0174	-0.0462	-6.70E-02
17	B	-0.1650	-0.0400	0.0258	0.0225	0.1065	1.00E-01
18	B	0.3342	0.0048	-0.0719	-0.0740	-0.2172	-4.61E-02
19	B	-0.3295	-0.0552	0.0499	0.0450	0.1933	3.61E-02
20	B	-0.0853	-0.0115	0.0396	0.0377	0.1644	-2.76E-02
21	B	-0.0631	-0.0608	0.0113	0.0073	0.0373	-1.45E-02
22	B	-0.2105	-0.0217	0.0366	0.0348	0.0410	2.08E-02
23	B	0.2602	0.0384	0.0013	-0.0003	0.0892	1.19E-04
24	B	-0.3431	-0.0736	0.0320	0.0266	0.1656	5.17E-02
25	B	-0.1633	-0.0539	0.0182	0.0149	0.1119	9.24E-02
26	B	-0.1431	-0.0305	0.0295	0.0262	0.1080	7.97E-02
27	B	-0.2633	-0.0351	0.0244	0.0210	0.0965	-1.11E-01
28	B	-0.3467	-0.0673	0.0368	0.0314	0.1583	3.83E-03
29	B	-0.1204	-0.0306	0.0103	0.0083	0.0139	3.42E-02
30	B	0.3065	-0.0042	-0.0623	-0.0650	-0.1828	1.82E-01
31	B	0.2805	0.0066	-0.0492	-0.0519	-0.1550	1.66E-01
32	B	0.3695	0.0450	-0.0259	-0.0274	-0.0386	-9.98E-02
33	B	-0.0370	-0.0515	0.0147	0.0107	0.0035	-1.24E-02
34	B	-0.2127	-0.0240	0.0356	0.0337	0.0448	2.37E-02
35	B	0.2477	0.0251	-0.0136	-0.0152	0.0635	1.70E-02
36	B	-0.0448	-0.0392	0.0010	-0.0022	-0.0042	1.24E-01
37	B	0.3102	0.0219	-0.0206	-0.0219	-0.0488	-6.25E-02
38	B	-0.3531	-0.0627	0.0479	0.0425	0.1880	2.94E-02
39	B	-0.2686	-0.0319	0.0287	0.0253	0.0792	-9.43E-02
40	Cl	-0.1105	0.7580	0.0023	0.1141	-0.5598	-6.18E-01
	F1(B39)	0.1105	-0.7580	-0.0023	-0.1141	0.5597	0.6579
	F2(Cl)	-0.1105	0.7580	0.0023	0.1141	-0.5598	-0.6181
	Total	0.0000	0.0000	0.0000	0.0000	0.0000	0.0398

Cl calibration curve constraction:

Charges on Cl	Cl 1s orbital energies
-2	-101.37098
-1	-101.51657
0	-101.75375
1	-102.07354
2	-102.47303

Orbital energies in Cl@B39 complex	
Cl 1s	-101.62826

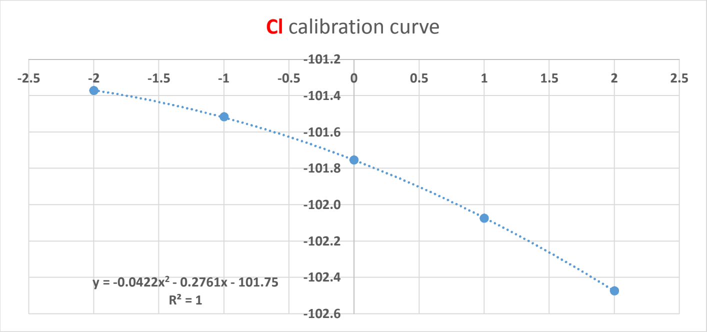

$$Q_{predicted} = -0.475$$

Br@B39 [0,1]		Mulliken Scheme	Lowdin Scheme	Hirshfeld Scheme	CM5 Scheme	NPA Scheme	Bader Scheme
Atom Number	Element	Charge density	Charge density	Charge density	Charge density	Charge density	Charge density
1	B	0.2947	0.0018	-0.0738	-0.0765	-0.1927	1.28E-02
2	B	-0.0235	-0.0473	-0.0067	-0.0127	-0.0101	1.02E-01
3	B	-0.0728	-0.0751	0.0064	-0.0012	0.0485	-1.95E-02
4	B	0.0956	0.0196	-0.0173	-0.0203	0.0610	9.43E-03
5	B	-0.2685	-0.0662	0.0316	0.0224	0.1320	6.01E-02
6	B	0.3300	0.0281	-0.0434	-0.0461	-0.0836	-6.74E-02
7	B	0.4004	0.0098	-0.0655	-0.0694	-0.2058	-6.82E-02
8	B	0.1643	0.0175	-0.0219	-0.0242	-0.0495	-6.38E-02
9	B	-0.3002	-0.0708	0.0425	0.0333	0.1694	2.34E-02
10	B	0.3392	0.0079	-0.0444	-0.0494	-0.1429	1.24E-01
11	B	-0.1701	-0.0427	0.0214	0.0150	0.0578	-1.05E-01
12	B	-0.0873	-0.0314	0.0316	0.0282	0.0383	1.93E-02
13	B	-0.0477	-0.0483	-0.0037	-0.0097	-0.0007	1.11E-01
14	B	-0.0367	-0.0244	0.0282	0.0244	0.1327	-2.49E-02
15	B	0.4067	0.0089	-0.0649	-0.0688	-0.1900	-6.09E-02
16	B	0.1609	0.0177	-0.0231	-0.0255	-0.0507	-6.39E-02
17	B	-0.2218	-0.0665	0.0112	0.0050	0.0944	9.11E-02
18	B	0.3649	-0.0035	-0.0784	-0.0822	-0.2144	-2.94E-02
19	B	-0.3203	-0.0681	0.0413	0.0321	0.1706	5.03E-03
20	B	-0.0647	-0.0365	0.0114	0.0077	0.0463	1.63E-02
21	B	-0.0411	-0.0657	0.0054	-0.0022	-0.0186	-2.42E-02
22	B	-0.1005	-0.0283	0.0320	0.0286	0.0272	2.03E-02
23	B	0.1298	0.0292	-0.0061	-0.0092	0.0816	-1.27E-02
24	B	-0.2909	-0.0854	0.0374	0.0274	0.1572	3.12E-02
25	B	-0.1848	-0.0532	0.0136	0.0074	0.0788	8.94E-02
26	B	-0.2184	-0.0533	0.0155	0.0093	0.0836	9.84E-02
27	B	-0.1527	-0.0443	0.0212	0.0148	0.0809	-1.48E-01
28	B	-0.2589	-0.0913	0.0228	0.0127	0.1382	3.24E-02
29	B	-0.0504	-0.0295	0.0164	0.0127	0.0526	2.70E-04
30	B	0.3239	-0.0151	-0.0758	-0.0808	-0.2028	1.88E-01
31	B	0.3311	-0.0036	-0.0574	-0.0624	-0.1592	1.54E-01
32	B	0.3069	0.0104	-0.0636	-0.0662	-0.1553	-2.55E-02
33	B	-0.0508	-0.0740	0.0071	-0.0005	0.0368	-1.32E-02
34	B	-0.0922	-0.0298	0.0334	0.0300	0.0434	1.55E-02
35	B	0.1013	0.0263	-0.0114	-0.0145	0.0648	1.18E-02
36	B	-0.0338	-0.0421	0.0007	-0.0052	-0.0084	9.00E-02
37	B	0.1525	0.0118	-0.0312	-0.0335	-0.0714	-5.45E-02
38	B	-0.2939	-0.0872	0.0293	0.0193	0.1355	-5.64E-03
39	B	-0.1939	-0.0467	0.0193	0.0129	0.0821	-1.15E-01
40	Br	-0.3261	1.1415	0.2090	0.4172	-0.2576	-3.57E-01
	F1(B39)	0.3261	-1.1415	-0.2091	-0.4172	0.2576	0.4047
	F2(Br)	-0.3261	1.1415	0.2090	0.4172	-0.2576	-0.3566
	Total	0.0000	0.0000	0.0000	0.0000	0.0000	0.0481

Br calibration curve constraction:

Charges on Br	Br 1s orbital energies
-2	-483.02043
-1	-483.11004
0	-483.29595
1	-483.55083
2	-483.85900

Orbital energies in Br@B39 complex

Br 1s -483.23916

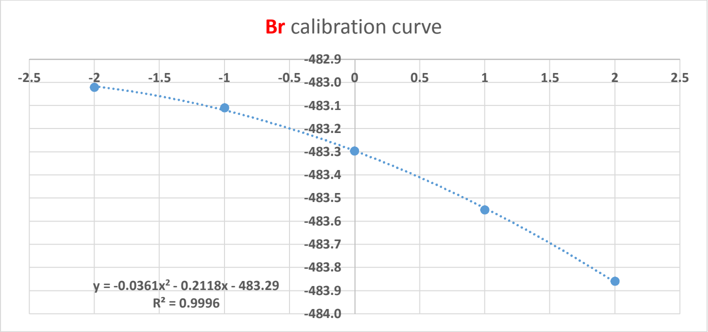

$Q_{predicted} = -0.251$

NO@B39 [0,1]		Mulliken Scheme	Lowdin Scheme	Hirshfeld Scheme	CM5 Scheme	NPA Scheme	Bader Scheme
Atom Number	Element	Charge density	Charge density	Charge density	Charge density	Charge density	Charge density
1	B	0.3144	0.0278	-0.0474	-0.0474	-0.0936	-6.65E-02
2	B	-0.0637	-0.0368	-0.0115	-0.0120	-0.0352	1.02E-01
3	B	-0.1195	-0.0633	0.0021	0.0020	0.0202	-1.62E-02
4	B	0.2371	0.0271	-0.0151	-0.0158	0.0665	-1.38E-02
5	B	-0.3697	-0.0423	0.0241	0.0237	0.1332	5.53E-03
6	B	0.3144	0.0278	-0.0475	-0.0474	-0.0929	-6.77E-02
7	B	0.3760	0.0049	-0.0774	-0.0775	-0.2009	-4.55E-02
8	B	0.2782	0.0166	-0.0286	-0.0292	-0.0471	-7.25E-02
9	B	-0.3697	-0.0423	0.0240	0.0236	0.1147	5.55E-03
10	B	0.3997	-0.0064	-0.0785	-0.0789	-0.1939	1.77E-01
11	B	-0.3405	-0.0346	0.0127	0.0118	0.0429	-1.43E-01
12	B	-0.2120	-0.0345	0.0265	0.0255	0.0295	1.02E-02
13	B	-0.0637	-0.0368	-0.0115	-0.0120	-0.0317	1.01E-01
14	B	-0.0525	-0.0315	0.0122	0.0124	0.0611	1.38E-02
15	B	0.3760	0.0049	-0.0774	-0.0775	-0.2036	-4.65E-02
16	B	0.2782	0.0166	-0.0286	-0.0293	-0.0521	-7.12E-02
17	B	-0.1452	-0.0522	0.0146	0.0146	0.0853	6.66E-02
18	B	0.3760	0.0049	-0.0773	-0.0775	-0.2100	-4.72E-02
19	B	-0.3697	-0.0423	0.0241	0.0237	0.1290	5.67E-03
20	B	-0.0525	-0.0315	0.0122	0.0123	0.0624	1.38E-02
21	B	-0.1195	-0.0633	0.0021	0.0020	0.0202	-1.64E-02
22	B	-0.2120	-0.0345	0.0266	0.0255	0.0271	1.02E-02
23	B	0.2371	0.0271	-0.0151	-0.0157	0.0666	-1.45E-02
24	B	-0.4504	-0.0557	0.0137	0.0132	0.1038	-1.24E-02
25	B	-0.1452	-0.0522	0.0146	0.0146	0.0839	6.66E-02
26	B	-0.1452	-0.0522	0.0146	0.0146	0.0878	6.67E-02
27	B	-0.3405	-0.0346	0.0127	0.0118	0.0383	-1.43E-01
28	B	-0.4504	-0.0557	0.0138	0.0133	0.1100	-1.26E-02
29	B	-0.0525	-0.0315	0.0122	0.0124	0.0621	1.37E-02
30	B	0.3997	-0.0064	-0.0786	-0.0789	-0.1923	1.77E-01
31	B	0.3997	-0.0064	-0.0785	-0.0789	-0.2103	1.77E-01
32	B	0.3144	0.0278	-0.0474	-0.0473	-0.0938	-6.80E-02
33	B	-0.1195	-0.0633	0.0021	0.0021	0.0170	-1.59E-02
34	B	-0.2120	-0.0345	0.0266	0.0255	0.0265	1.01E-02
35	B	0.2371	0.0271	-0.0151	-0.0157	0.0715	-1.33E-02
36	B	-0.0637	-0.0368	-0.0115	-0.0120	-0.0308	1.01E-01
37	B	0.2782	0.0166	-0.0286	-0.0292	-0.0527	-7.18E-02
38	B	-0.4504	-0.0557	0.0138	0.0133	0.1110	-1.25E-02
39	B	-0.3405	-0.0346	0.0127	0.0118	0.0432	-1.43E-01
40	O	0.2170	0.4615	0.1731	0.2507	-0.2357	-4.47E-01
41	N	0.2274	0.3810	0.2853	0.2223	0.3625	4.77E-01
	F1(B39)	-0.4444	-0.8425	-0.4577	-0.4722	-0.1268	0.0095
	F2(NO)	0.4444	0.8425	0.4585	0.4730	0.1268	0.0297
	Total	0.0000	0.0000	0.0008	0.0008	0.0000	0.0392

N+O calibration curve constraction:

Charges on NO	O 1s orbital energies	N 1s orbital energies
-2	-18.92336	-14.07920
-1	-19.10629	-14.26065
0	-19.34540	-14.51986
1	-19.66771	-14.86562
2	-20.02529	-15.28306

Orbital energies in NO@B39 complex	
O 1s	-19.41203
N 1s	-14.60064

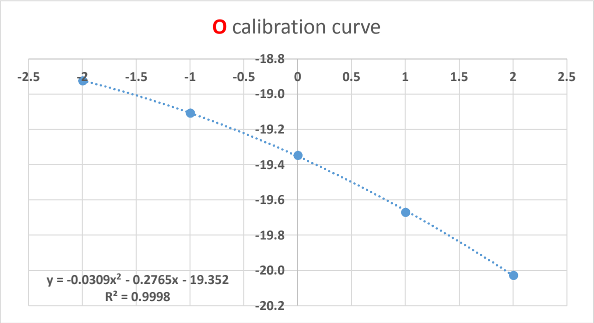

$Q^{O}_{predicted} = 0.212$

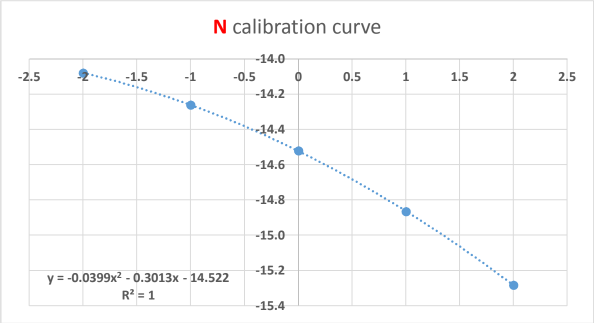

$Q^{N}_{predicted} = 0.253$

CN@B39 [0,1]		Mulliken Scheme	Lowdin Scheme	Hirshfeld Scheme	CM5 Scheme	NPA Scheme	Bader Scheme
Atom Number	Element	Charge density	Charge density	Charge density	Charge density	Charge density	Charge density
1	B	0.1781	0.0118	-0.0759	-0.0759	-0.2013	5.43E-02
2	B	-0.0573	-0.0242	-0.0040	-0.0060	-0.0488	1.36E-01
3	B	-0.1132	-0.0454	0.0179	0.0171	0.0651	1.35E-03
4	B	0.1468	0.0352	-0.0042	-0.0064	0.0868	-1.09E-02
5	B	-0.1237	-0.0107	0.0500	0.0481	0.1745	5.01E-02
6	B	0.1781	0.0118	-0.0758	-0.0758	-0.2024	5.43E-02
7	B	0.3409	0.0312	-0.0530	-0.0536	-0.1959	-5.40E-02
8	B	0.0740	0.0178	-0.0346	-0.0368	-0.1080	-1.50E-02
9	B	-0.1237	-0.0107	0.0500	0.0482	0.1692	5.00E-02
10	B	0.2959	0.0118	-0.0697	-0.0710	-0.2394	2.20E-01
11	B	-0.1545	-0.0012	0.0596	0.0565	0.2121	-1.61E-01
12	B	-0.0125	-0.0242	0.0461	0.0423	0.1219	3.21E-02
13	B	-0.0573	-0.0242	-0.0039	-0.0060	-0.0491	1.36E-01
14	B	-0.0316	-0.0195	0.0354	0.0354	0.1411	-2.23E-02
15	B	0.3409	0.0312	-0.0531	-0.0536	-0.1897	-5.44E-02
16	B	0.0740	0.0178	-0.0346	-0.0368	-0.1133	-1.85E-02
17	B	-0.1433	-0.0178	0.0410	0.0406	0.1421	7.12E-02
18	B	0.3409	0.0312	-0.0530	-0.0536	-0.1887	-5.22E-02
19	B	-0.1237	-0.0107	0.0500	0.0482	0.1711	5.00E-02
20	B	-0.0316	-0.0195	0.0355	0.0355	0.1411	-2.21E-02
21	B	-0.1132	-0.0454	0.0179	0.0171	0.0594	1.38E-03
22	B	-0.0125	-0.0242	0.0461	0.0423	0.1216	3.19E-02
23	B	0.1468	0.0352	-0.0043	-0.0064	0.0865	-1.10E-02
24	B	-0.1354	-0.0188	0.0508	0.0488	0.2100	1.44E-02
25	B	-0.1433	-0.0178	0.0410	0.0406	0.1455	7.11E-02
26	B	-0.1433	-0.0178	0.0409	0.0406	0.1432	7.10E-02
27	B	-0.1545	-0.0012	0.0596	0.0565	0.2123	-1.61E-01
28	B	-0.1354	-0.0188	0.0508	0.0488	0.2030	1.43E-02
29	B	-0.0316	-0.0195	0.0354	0.0354	0.1399	-2.24E-02
30	B	0.2959	0.0118	-0.0697	-0.0710	-0.2319	2.19E-01
31	B	0.2959	0.0118	-0.0697	-0.0710	-0.2315	2.20E-01
32	B	0.1781	0.0118	-0.0759	-0.0759	-0.2018	5.35E-02
33	B	-0.1132	-0.0454	0.0178	0.0170	0.0609	1.24E-03
34	B	-0.0125	-0.0242	0.0461	0.0423	0.1188	3.21E-02
35	B	0.1468	0.0352	-0.0043	-0.0065	0.0895	-8.44E-03
36	B	-0.0573	-0.0242	-0.0040	-0.0060	-0.0547	1.36E-01
37	B	0.0740	0.0178	-0.0346	-0.0367	-0.1058	-1.93E-02
38	B	-0.1354	-0.0188	0.0508	0.0488	0.2112	1.44E-02
39	B	-0.1545	-0.0012	0.0597	0.0566	0.2058	-1.61E-01
40	C	-0.8426	-0.0595	-0.0455	0.1361	0.1672	6.89E-01
41	N	0.0500	0.2216	-0.1319	-0.2529	-1.2375	-1.59E+00
	F1(B39)	0.7926	-0.1621	0.1780	0.1174	1.0703	0.9427
	F2(CN)	-0.7926	0.1621	-0.1774	-0.1168	-1.0703	-0.8992
	Total	0.0000	0.0000	0.0006	0.0006	0.0000	0.0436

N+C calibration curve constraction:

Charges on CN	N 1s orbital energies	C 1s orbital energies
-2	-14.14861	-10.02157
-1	-14.26022	-10.11569
0	-14.44832	-10.34491
1	-14.72820	-10.63958
2	-15.05867	-10.99190

Orbital energies in CN@B39 complex	
N 1s	-14.30035
C 1s	-10.16208

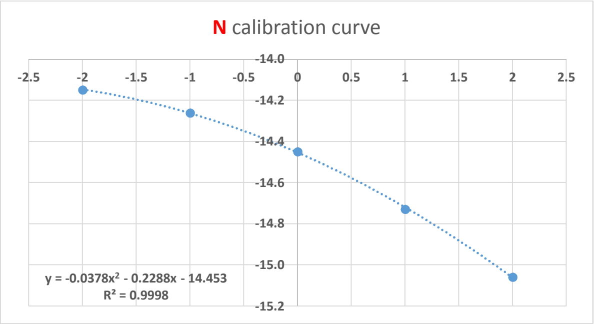

$Q^{N}_{predicted} = -0.763$

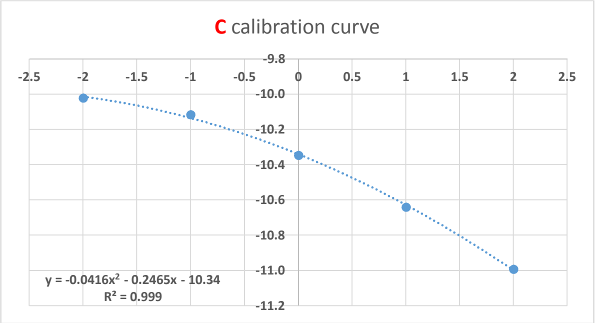

$Q^{C}_{predicted} = -0.841$

FO@B39 [0,1]		Mulliken Scheme	Lowdin Scheme	Hirshfeld Scheme	CM5 Scheme	NPA Scheme	Bader Scheme
Atom Number	Element	Charge density	Charge density	Charge density	Charge density	Charge density	Charge density
1	B	0.2678	0.0281	-0.0473	-0.0488	-0.1054	-2.06E-02
2	B	-0.0174	-0.0191	0.0042	0.0031	0.0089	1.14E-01
3	B	-0.0884	-0.0413	0.0108	0.0087	0.0198	-2.14E-02
4	B	0.1817	0.0293	-0.0120	-0.0128	0.0719	1.79E-02
5	B	-0.2005	-0.0192	0.0398	0.0381	0.1448	5.47E-02
6	B	0.2951	0.0559	-0.0158	-0.0172	-0.0155	-1.44E-01
7	B	0.2288	0.0089	-0.0763	-0.0771	-0.2363	-3.56E-02
8	B	0.1909	0.0155	-0.0303	-0.0311	-0.0722	-3.17E-02
9	B	-0.2087	-0.0188	0.0416	0.0399	0.1588	2.42E-02
10	B	0.2101	0.0182	-0.0509	-0.0518	-0.1651	1.74E-01
11	B	-0.1984	-0.0199	0.0301	0.0287	0.1038	-9.59E-02
12	B	-0.0984	-0.0289	0.0355	0.0340	0.0581	1.73E-02
13	B	-0.0349	-0.0182	0.0040	0.0029	-0.0021	1.11E-01
14	B	-0.0221	-0.0208	0.0339	0.0314	0.1534	-1.63E-02
15	B	0.2471	0.0132	-0.0709	-0.0718	-0.2269	-3.14E-02
16	B	0.1917	0.0119	-0.0345	-0.0354	-0.0749	-3.25E-02
17	B	-0.1304	-0.0364	0.0211	0.0187	0.1083	8.06E-02
18	B	0.2349	0.0124	-0.0682	-0.0691	-0.2176	-4.51E-02
19	B	-0.1972	-0.0140	0.0366	0.0348	0.1343	1.96E-02
20	B	-0.0539	-0.0321	0.0010	-0.0014	-0.0289	4.49E-02
21	B	-0.0930	-0.0356	0.0169	0.0148	0.0058	7.76E-03
22	B	-0.0995	-0.0287	0.0356	0.0341	0.0560	2.06E-02
23	B	0.1949	0.0306	-0.0123	-0.0132	0.0673	1.87E-02
24	B	-0.2336	-0.0203	0.0438	0.0419	0.1828	8.03E-03
25	B	-0.1315	-0.0147	0.0398	0.0375	0.1233	6.31E-02
26	B	-0.1426	-0.0382	0.0246	0.0223	0.1154	8.36E-02
27	B	-0.1771	-0.0134	0.0397	0.0384	0.1362	-1.58E-01
28	B	-0.2189	-0.0257	0.0401	0.0382	0.1804	4.35E-02
29	B	-0.0307	-0.0099	0.0301	0.0277	0.0972	-2.43E-02
30	B	0.1688	0.0069	-0.0689	-0.0698	-0.2134	2.15E-01
31	B	0.1872	0.0096	-0.0636	-0.0645	-0.1955	1.82E-01
32	B	0.2566	-0.0019	-0.0845	-0.0859	-0.2234	3.77E-02
33	B	-0.0684	-0.0455	0.0131	0.0110	0.0566	-5.21E-03
34	B	-0.0912	-0.0272	0.0357	0.0342	0.0529	1.58E-02
35	B	0.1949	0.0370	-0.0037	-0.0046	0.0826	7.10E-03
36	B	-0.0470	-0.0148	0.0033	0.0022	-0.0058	9.67E-02
37	B	0.1918	0.0131	-0.0327	-0.0335	-0.0734	-3.46E-02
38	B	-0.2169	-0.0264	0.0340	0.0320	0.1583	1.71E-02
39	B	-0.1746	-0.0165	0.0298	0.0285	0.1118	-1.16E-01
40	F	-0.0284	0.2473	0.0715	0.1227	-0.1761	-3.01E-01
41	O	-0.2388	0.0496	-0.0444	-0.0372	-0.3561	-3.18E-01
	F1(B39)	0.2672	-0.2969	-0.0263	-0.0848	0.5322	0.6618
	F2(FO)	-0.2672	0.2969	0.0270	0.0855	-0.5322	-0.6192
	Total	0.0000	0.0000	0.0007	0.0007	0.0000	0.0426

F+O calibration curve constraction:

Charges on FO	F 1s orbital energies	O 1s orbital energies
-2	-24.42790	-18.82396
-1	-24.63991	-19.03829
0	-24.87414	-19.37617
1	-25.21402	-19.75222
2	-25.60464	-20.20892

Orbital energies in FO@B39 complex	
F 1s	-24.81417
O 1s	-19.24582

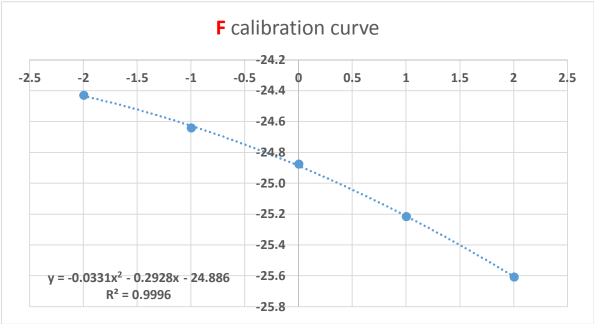

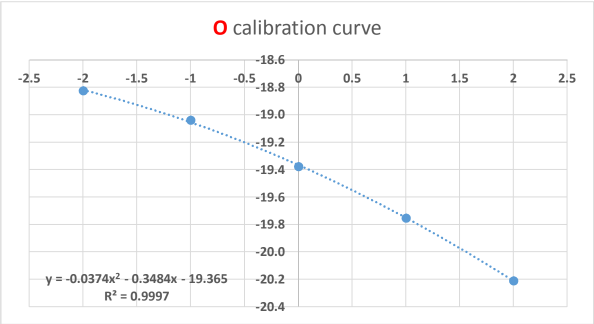

$Q^{F}_{predicted} = -0.253$

$Q^{O}_{predicted} = -0.355$

CF@B39 [0,1]		Mulliken Scheme	Lowdin Scheme	Hirshfeld Scheme	CM5 Scheme	NPA Scheme	Bader Scheme
Atom Number	Element	Charge density	Charge density	Charge density	Charge density	Charge density	Charge density
1	B	0.1922	0.0246	-0.0526	-0.0554	-0.1061	-5.84E-02
2	B	-0.0395	-0.0336	-0.0109	-0.0129	-0.0239	9.94E-02
3	B	-0.0168	-0.0666	0.0044	0.0002	0.0375	-3.00E-02
4	B	0.1659	0.0272	-0.0167	-0.0181	0.0561	-9.02E-03
5	B	-0.1957	-0.0418	0.0254	0.0219	0.1366	1.89E-03
6	B	0.1922	0.0246	-0.0526	-0.0554	-0.1061	-5.68E-02
7	B	0.2673	0.0078	-0.0748	-0.0766	-0.1986	-5.26E-02
8	B	0.2112	0.0157	-0.0299	-0.0312	-0.0572	-6.80E-02
9	B	-0.1957	-0.0418	0.0253	0.0218	0.1366	1.90E-03
10	B	0.2938	-0.0044	-0.0782	-0.0800	-0.2001	1.71E-01
11	B	-0.2829	-0.0306	0.0124	0.0100	0.0448	-1.38E-01
12	B	-0.1985	-0.0345	0.0257	0.0235	0.0265	5.16E-03
13	B	-0.0395	-0.0336	-0.0109	-0.0129	-0.0239	9.92E-02
14	B	-0.0226	-0.0393	0.0097	0.0050	0.0760	1.79E-02
15	B	0.2673	0.0078	-0.0747	-0.0765	-0.1986	-5.20E-02
16	B	0.2112	0.0157	-0.0300	-0.0313	-0.0572	-6.82E-02
17	B	-0.0600	-0.0589	0.0147	0.0101	0.0901	6.52E-02
18	B	0.2673	0.0078	-0.0748	-0.0765	-0.1986	-5.31E-02
19	B	-0.1957	-0.0418	0.0255	0.0220	0.1366	1.85E-03
20	B	-0.0226	-0.0393	0.0097	0.0049	0.0760	1.78E-02
21	B	-0.0168	-0.0666	0.0045	0.0002	0.0375	-2.99E-02
22	B	-0.1985	-0.0345	0.0258	0.0235	0.0265	5.10E-03
23	B	0.1659	0.0272	-0.0167	-0.0181	0.0561	-9.48E-03
24	B	-0.3191	-0.0540	0.0135	0.0096	0.1183	-1.80E-02
25	B	-0.0600	-0.0589	0.0147	0.0101	0.0901	6.52E-02
26	B	-0.0600	-0.0589	0.0147	0.0101	0.0901	6.53E-02
27	B	-0.2829	-0.0306	0.0124	0.0100	0.0448	-1.38E-01
28	B	-0.3191	-0.0540	0.0135	0.0097	0.1183	-1.82E-02
29	B	-0.0226	-0.0393	0.0098	0.0050	0.0760	1.77E-02
30	B	0.2938	-0.0044	-0.0783	-0.0801	-0.2001	1.71E-01
31	B	0.2938	-0.0044	-0.0783	-0.0801	-0.2001	1.71E-01
32	B	0.1922	0.0246	-0.0526	-0.0554	-0.1061	-5.81E-02
33	B	-0.0168	-0.0666	0.0045	0.0002	0.0375	-2.98E-02
34	B	-0.1985	-0.0345	0.0258	0.0236	0.0265	4.96E-03
35	B	0.1659	0.0272	-0.0166	-0.0180	0.0561	-9.24E-03
36	B	-0.0395	-0.0336	-0.0108	-0.0129	-0.0239	9.93E-02
37	B	0.2112	0.0157	-0.0299	-0.0312	-0.0572	-6.79E-02
38	B	-0.3191	-0.0540	0.0135	0.0097	0.1183	-1.80E-02
39	B	-0.2829	-0.0306	0.0123	0.0099	0.0448	-1.38E-01
40	C	-0.1074	0.3372	0.3282	0.4286	0.5243	8.29E-01
41	F	0.1210	0.5281	0.1439	0.1534	-0.5235	-7.43E-01
	F1(B39)	-0.0135	-0.8653	-0.4717	-0.5816	-0.0008	-0.0411
	F2(CF)	0.0135	0.8653	0.4721	0.5820	0.0008	0.0862
	Total	0.0000	0.0000	0.0005	0.0005	0.0000	0.0451

F+C calibration curve constraction:

Charges on CF	F 1s orbital energies	C 1s orbital energies
-2	-24.60253	-10.03886
-1	-24.72069	-10.16461
0	-24.87854	-10.37149
1	-25.12975	-10.71409
2	-25.41892	-11.12917

Orbital energies in CF@B39 complex	
F 1s	-24.92486
C 1s	-10.45571

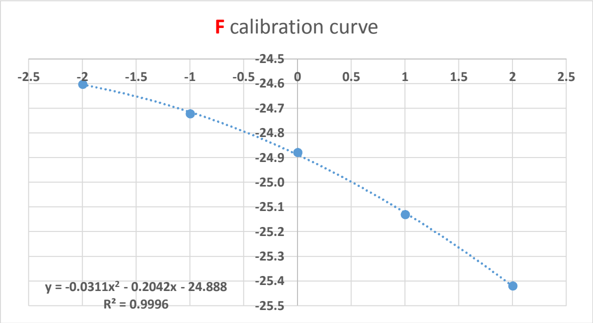

$Q^{F}_{predicted} = 0.176$

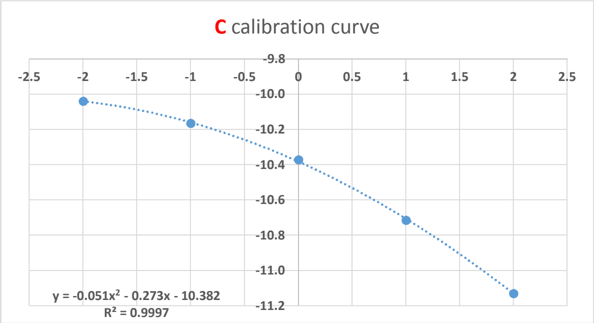

$Q^{C}_{predicted} = 0.257$

**Cu@B39 [0,1]**		Mulliken Scheme	Lowdin Scheme	Hirshfeld Scheme	CM5 Scheme	NPA Scheme	Bader Scheme
Atom Number	Element	Charge density	Charge density	Charge density	Charge density	Charge density	Charge density
1	B	0.2796	0.0151	-0.0758	-0.0802	-0.1689	-1.41E-02
2	B	-0.0214	-0.0243	-0.0119	-0.0215	-0.0456	7.33E-02
3	B	-0.0726	-0.0381	0.0071	-0.0052	0.0223	-6.73E-02
4	B	0.1881	0.0382	-0.0154	-0.0203	0.0685	-2.24E-02
5	B	-0.3339	-0.0233	0.0302	0.0153	0.0875	-2.13E-02
6	B	0.2811	0.0151	-0.0758	-0.0802	-0.1699	-1.19E-02
7	B	0.3700	0.0250	-0.0700	-0.0763	-0.1806	-5.61E-02
8	B	0.2836	0.0263	-0.0292	-0.0330	-0.0550	-7.41E-02
9	B	-0.3298	-0.0232	0.0302	0.0153	0.0890	-2.12E-02
10	B	0.2451	0.0132	-0.0710	-0.0791	-0.1887	1.48E-01
11	B	-0.2477	-0.0190	0.0123	0.0019	0.0180	-1.68E-01
12	B	-0.1839	-0.0183	0.0267	0.0211	0.0127	-7.99E-03
13	B	-0.0184	-0.0243	-0.0118	-0.0215	-0.0462	7.32E-02
14	B	-0.0647	-0.0229	0.0097	0.0037	0.0640	-2.53E-02
15	B	0.3714	0.0250	-0.0700	-0.0763	-0.1740	-5.75E-02
16	B	0.2834	0.0264	-0.0292	-0.0330	-0.0504	-7.41E-02
17	B	-0.1700	-0.0358	0.0085	-0.0016	0.0462	5.49E-02
18	B	0.3688	0.0250	-0.0699	-0.0762	-0.1742	-5.83E-02
19	B	-0.3299	-0.0232	0.0303	0.0154	0.0910	-2.13E-02
20	B	-0.0658	-0.0229	0.0098	0.0037	0.0638	-2.51E-02
21	B	-0.0724	-0.0381	0.0071	-0.0052	0.0178	-6.72E-02
22	B	-0.1875	-0.0183	0.0266	0.0211	0.0142	-8.13E-03
23	B	0.1907	0.0382	-0.0154	-0.0204	0.0665	-2.08E-02
24	B	-0.3355	-0.0385	0.0166	0.0002	0.0588	-4.53E-02
25	B	-0.1766	-0.0358	0.0084	-0.0017	0.0496	5.48E-02
26	B	-0.1731	-0.0358	0.0084	-0.0016	0.0474	5.49E-02
27	B	-0.2490	-0.0190	0.0123	0.0019	0.0219	-1.68E-01
28	B	-0.3347	-0.0385	0.0166	0.0003	0.0530	-4.51E-02
29	B	-0.0658	-0.0229	0.0096	0.0036	0.0627	-2.54E-02
30	B	0.2425	0.0132	-0.0710	-0.0791	-0.1811	1.49E-01
31	B	0.2480	0.0132	-0.0710	-0.0791	-0.1724	1.49E-01
32	B	0.2816	0.0152	-0.0759	-0.0802	-0.1695	-1.58E-02
33	B	-0.0734	-0.0381	0.0070	-0.0052	0.0183	-6.72E-02
34	B	-0.1874	-0.0183	0.0267	0.0211	0.0146	-8.00E-03
35	B	0.1909	0.0383	-0.0154	-0.0204	0.0668	-2.12E-02
36	B	-0.0206	-0.0243	-0.0119	-0.0215	-0.0494	7.34E-02
37	B	0.2853	0.0263	-0.0292	-0.0330	-0.0534	-7.35E-02
38	B	-0.3362	-0.0385	0.0166	0.0002	0.0573	-4.55E-02
39	B	-0.2469	-0.0190	0.0124	0.0019	0.0136	-1.68E-01
40	Cu	0.1872	0.3064	0.4871	0.8255	0.7541	7.19E-01
	F1(B39)	-0.1872	-0.3064	-0.4869	-0.8253	-0.7541	-0.6760
	F2(Cu)	0.1872	0.3064	0.4871	0.8255	0.7541	0.7191
	Total	0.0000	0.0000	0.0001	0.0001	0.0000	0.0431

Cu calibration curve constraction:

Charges on Cu	Cu 1s orbital energies	Cu 2s orbital energies	Cu 3s orbital energies
-2	-323.17838	-38.77958	-4.19666
-1	-323.22079	-38.81717	-4.23776
0	-323.30118	-38.89197	-4.31710
1	-323.44751	-39.03023	-4.46189
2	-323.90611	-39.52010	-4.95466

Orbital energies in Cu@B39 complex	
Cu 1s	-323.36111
Cu 2s	-38.94670
Cu 3s	-4.37551

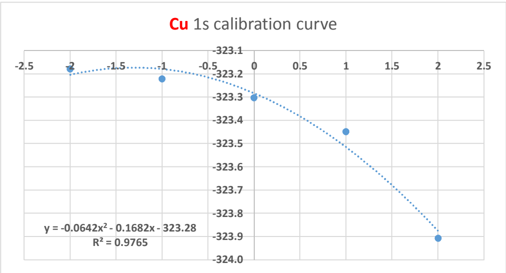

$Q_{predicted} = 0.416$

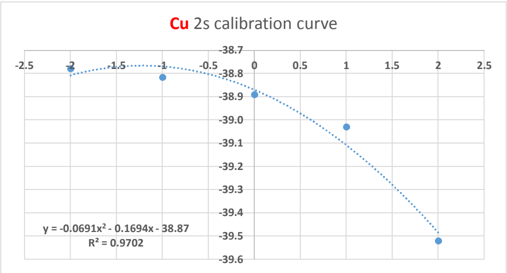

$Q_{predicted} = 0.391$

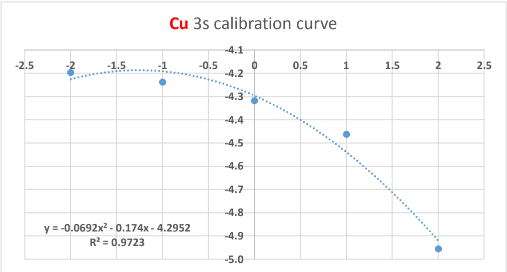

$Q_{predicted} = 0.398$

Ag@B39 [0,1]		Mulliken Scheme	Lowdin Scheme	Hirshfeld Scheme	CM5 Scheme	NPA Scheme	Bader Scheme
Atom Number	Element	Charge density	Charge density	Charge density	Charge density	Charge density	Charge density
1	B	0.3475	0.0105	-0.0769	-0.0831	-0.1713	-8.11E-03
2	B	-0.0539	-0.0336	-0.0124	-0.0260	-0.0442	7.58E-02
3	B	-0.1483	-0.0487	0.0066	-0.0108	0.0210	-6.51E-02
4	B	0.1399	0.0336	-0.0152	-0.0223	0.0644	-2.10E-02
5	B	-0.3339	-0.0358	0.0298	0.0087	0.0833	-1.87E-02
6	B	0.3475	0.0105	-0.0769	-0.0830	-0.1732	-6.04E-03
7	B	0.4576	0.0193	-0.0708	-0.0796	-0.1823	-4.61E-02
8	B	0.2531	0.0224	-0.0295	-0.0348	-0.0503	-7.00E-02
9	B	-0.3339	-0.0358	0.0299	0.0087	0.0968	-1.87E-02
10	B	0.3345	0.0057	-0.0719	-0.0833	-0.1831	1.59E-01
11	B	-0.2365	-0.0287	0.0120	-0.0027	0.0197	-1.68E-01
12	B	-0.1437	-0.0245	0.0263	0.0184	0.0147	-7.58E-03
13	B	-0.0539	-0.0336	-0.0124	-0.0260	-0.0435	7.57E-02
14	B	-0.0756	-0.0294	0.0091	0.0005	0.0651	-2.51E-02
15	B	0.4576	0.0193	-0.0708	-0.0797	-0.1748	-4.79E-02
16	B	0.2531	0.0224	-0.0294	-0.0348	-0.0556	-7.00E-02
17	B	-0.2376	-0.0449	0.0080	-0.0063	0.0481	5.58E-02
18	B	0.4576	0.0193	-0.0707	-0.0796	-0.1756	-4.86E-02
19	B	-0.3339	-0.0358	0.0299	0.0088	0.0896	-1.85E-02
20	B	-0.0756	-0.0294	0.0091	0.0006	0.0648	-2.50E-02
21	B	-0.1483	-0.0487	0.0066	-0.0108	0.0257	-6.50E-02
22	B	-0.1437	-0.0245	0.0262	0.0183	0.0151	-7.74E-03
23	B	0.1399	0.0336	-0.0153	-0.0223	0.0641	-1.98E-02
24	B	-0.3504	-0.0523	0.0163	-0.0067	0.0564	-4.27E-02
25	B	-0.2376	-0.0449	0.0079	-0.0064	0.0504	5.59E-02
26	B	-0.2376	-0.0449	0.0079	-0.0063	0.0490	5.57E-02
27	B	-0.2365	-0.0287	0.0120	-0.0027	0.0188	-1.68E-01
28	B	-0.3504	-0.0523	0.0164	-0.0067	0.0521	-4.29E-02
29	B	-0.0756	-0.0294	0.0090	0.0005	0.0640	-2.52E-02
30	B	0.3345	0.0057	-0.0719	-0.0833	-0.1762	1.60E-01
31	B	0.3345	0.0057	-0.0719	-0.0833	-0.1739	1.60E-01
32	B	0.3475	0.0105	-0.0769	-0.0831	-0.1719	-9.58E-03
33	B	-0.1483	-0.0487	0.0065	-0.0108	0.0216	-6.50E-02
34	B	-0.1437	-0.0245	0.0262	0.0183	0.0133	-7.71E-03
35	B	0.1399	0.0336	-0.0153	-0.0223	0.0629	-1.95E-02
36	B	-0.0539	-0.0336	-0.0124	-0.0260	-0.0487	7.57E-02
37	B	0.2531	0.0224	-0.0294	-0.0348	-0.0490	-6.95E-02
38	B	-0.3504	-0.0523	0.0164	-0.0067	0.0586	-4.27E-02
39	B	-0.2365	-0.0287	0.0121	-0.0027	0.0164	-1.68E-01
40	Ag	0.1418	0.6196	0.5059	0.9843	0.7376	5.86E-01
	F1(B39)	-0.1418	-0.6196	-0.5058	-0.9843	-0.7376	-0.5444
	F2(Ag)	0.1418	0.6196	0.5059	0.9843	0.7376	0.5861
	Total	0.0000	0.0000	0.0001	0.0001	0.0000	0.0418

Ag calibration curve constraction:

Charges on Ag	Ag 4s orbital energies
-2	-3.67322
-1	-3.67644
0	-3.75852
1	-3.89155
2	-4.27212

Orbital energies in Ag@B39 complex	
Ag 4s	-3.81311

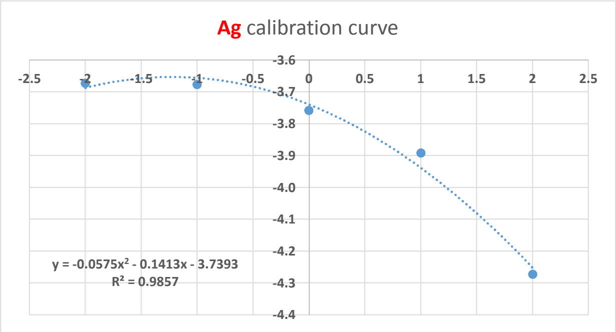

$Q_{predicted} = 0.443$

**Au@B39 [0,1]**		Mulliken Scheme	Lowdin Scheme	Hirshfeld Scheme	CM5 Scheme	NPA Scheme	Bader Scheme
Atom Number	Element	Charge density	Charge density	Charge density	Charge density	Charge density	Charge density
1	B	0.3406	0.0089	-0.0811	-0.0865	-0.1838	5.42E-04
2	B	-0.0424	-0.0365	-0.0142	-0.0259	-0.0472	7.73E-02
3	B	-0.1203	-0.0514	0.0068	-0.0082	0.0212	-6.52E-02
4	B	0.1307	0.0332	-0.0168	-0.0229	0.0641	-1.81E-02
5	B	-0.3016	-0.0412	0.0301	0.0119	0.0921	-1.39E-02
6	B	0.3385	0.0089	-0.0811	-0.0864	-0.1911	2.72E-03
7	B	0.4507	0.0180	-0.0739	-0.0816	-0.1915	-3.97E-02
8	B	0.2016	0.0220	-0.0321	-0.0367	-0.0613	-6.09E-02
9	B	-0.3001	-0.0412	0.0302	0.0120	0.0987	-1.36E-02
10	B	0.3613	0.0065	-0.0718	-0.0817	-0.1843	1.57E-01
11	B	-0.1681	-0.0319	0.0115	-0.0012	0.0186	-1.63E-01
12	B	-0.0955	-0.0249	0.0250	0.0182	0.0199	-3.17E-03
13	B	-0.0391	-0.0365	-0.0141	-0.0259	-0.0465	7.72E-02
14	B	-0.0763	-0.0306	0.0074	0.0000	0.0709	-2.07E-02
15	B	0.4485	0.0180	-0.0740	-0.0816	-0.1844	-4.16E-02
16	B	0.2028	0.0220	-0.0320	-0.0366	-0.0625	-6.00E-02
17	B	-0.2170	-0.0484	0.0064	-0.0059	0.0498	5.89E-02
18	B	0.4482	0.0180	-0.0739	-0.0815	-0.1842	-4.25E-02
19	B	-0.3003	-0.0412	0.0302	0.0120	0.0878	-1.38E-02
20	B	-0.0746	-0.0306	0.0074	0.0000	0.0721	-2.04E-02
21	B	-0.1178	-0.0514	0.0068	-0.0082	0.0299	-6.52E-02
22	B	-0.0987	-0.0249	0.0250	0.0181	0.0211	-3.29E-03
23	B	0.1316	0.0332	-0.0169	-0.0229	0.0681	-1.82E-02
24	B	-0.2644	-0.0587	0.0165	-0.0034	0.0521	-3.45E-02
25	B	-0.2235	-0.0485	0.0063	-0.0060	0.0531	5.88E-02
26	B	-0.2199	-0.0485	0.0063	-0.0060	0.0508	5.88E-02
27	B	-0.1699	-0.0319	0.0115	-0.0012	0.0198	-1.63E-01
28	B	-0.2643	-0.0587	0.0165	-0.0034	0.0479	-3.44E-02
29	B	-0.0747	-0.0306	0.0073	-0.0001	0.0708	-2.07E-02
30	B	0.3583	0.0065	-0.0718	-0.0817	-0.1770	1.58E-01
31	B	0.3615	0.0065	-0.0718	-0.0817	-0.1755	1.58E-01
32	B	0.3396	0.0089	-0.0812	-0.0865	-0.1843	-6.84E-04
33	B	-0.1204	-0.0514	0.0067	-0.0082	0.0202	-6.53E-02
34	B	-0.0992	-0.0249	0.0250	0.0182	0.0195	-3.16E-03
35	B	0.1331	0.0333	-0.0169	-0.0229	0.0635	-1.66E-02
36	B	-0.0420	-0.0365	-0.0142	-0.0259	-0.0511	7.73E-02
37	B	0.2028	0.0220	-0.0320	-0.0366	-0.0548	-6.08E-02
38	B	-0.2648	-0.0587	0.0165	-0.0034	0.0547	-3.44E-02
39	B	-0.1667	-0.0319	0.0116	-0.0011	0.0135	-1.63E-01
40	Au	-0.5885	0.7055	0.5596	0.9720	0.7995	4.17E-01
	F1(B39)	0.5885	-0.7055	-0.5592	-0.9717	-0.7995	-0.3758
	F2(Au)	-0.5885	0.7055	0.5596	0.9720	0.7995	0.4170
	Total	0.0000	0.0000	0.0004	0.0004	0.0000	0.0412

Au calibration curve constraction:

Charges on Au	Au 5s orbital energies
-2	-4.05093
-1	-4.06707
0	-4.16613
1	-4.30664
2	-4.6373

Orbital energies in Au@B39 complex

Au 5s	-4.22134

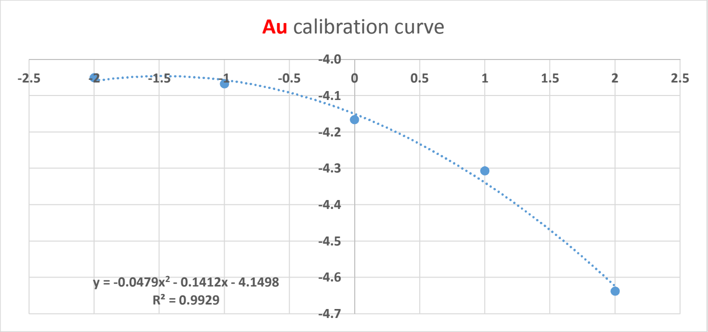

$$Q_{predicted} = 0.441$$

Sc3N@B39 [0,1]		Mulliken Scheme	Lowdin Scheme	Hirshfeld Scheme	CM5 Scheme	NPA Scheme	Bader Scheme
Atom Number	Element	Charge density	Charge density	Charge density	Charge density	Charge density	Charge density
1	C	0.0179	-0.0106	-0.0109	-0.0175	-0.0188	
2	C	-0.0788	-0.0118	0.0099	-0.0001	-0.0004	
3	C	0.0976	-0.0038	-0.0159	-0.0472	-0.1062	
4	C	0.0483	-0.0021	-0.0093	-0.0334	-0.0767	
5	C	-0.0137	-0.0149	-0.0245	-0.0259	-0.0435	
6	C	-0.0982	-0.0140	-0.0196	-0.0226	-0.0343	
7	C	0.0004	-0.0102	0.0116	0.0094	0.0439	
8	C	-0.1586	-0.0115	-0.0171	-0.0225	-0.0311	
9	C	-0.1765	-0.0144	-0.0178	-0.0229	-0.0276	
10	C	-0.0603	-0.0154	0.0058	0.0012	0.0147	
11	C	-0.0213	-0.0037	-0.0082	-0.0267	-0.0647	
12	C	-0.0565	-0.0131	-0.0177	-0.0204	-0.0167	
13	C	0.1029	-0.0070	-0.0142	-0.0346	-0.0786	
14	C	0.0544	-0.0080	-0.0329	-0.0804	-0.1459	
15	C	0.0636	-0.0169	0.0029	-0.0260	-0.0533	
16	C	-0.0376	-0.0081	-0.0152	-0.0225	-0.0397	
17	C	0.0251	-0.0061	-0.0130	-0.0261	-0.0598	
18	C	0.0597	-0.0207	-0.0059	-0.0446	-0.0825	
19	C	0.1016	-0.0084	-0.0171	-0.0409	-0.0921	
20	C	0.1155	-0.0074	-0.0377	-0.0906	-0.1448	
21	C	0.0967	-0.0072	-0.0141	-0.0344	-0.0819	
22	C	0.0634	-0.0167	0.0032	-0.0253	-0.0543	
23	C	0.0458	-0.0022	-0.0094	-0.0338	-0.0773	
24	C	0.0562	-0.0080	-0.0327	-0.0798	-0.1445	
25	C	0.1054	-0.0084	-0.0170	-0.0410	-0.0963	
26	C	0.0324	-0.0060	-0.0130	-0.0263	-0.0588	
27	C	0.0596	-0.0208	-0.0061	-0.0451	-0.0814	
28	C	0.1139	-0.0074	-0.0378	-0.0908	-0.1449	
29	C	-0.0147	-0.0150	-0.0245	-0.0260	-0.0429	
30	C	-0.0027	-0.0103	0.0117	0.0094	0.0421	
31	C	-0.1568	-0.0115	-0.0170	-0.0224	-0.0323	
32	C	-0.1001	-0.0141	-0.0195	-0.0226	-0.0343	
33	C	-0.1618	-0.0115	-0.0170	-0.0224	-0.0303	
34	C	-0.0812	-0.0119	0.0099	0.0000	0.0002	
35	C	0.0636	-0.0162	-0.0213	-0.0251	-0.0297	
36	C	0.0946	-0.0038	-0.0157	-0.0468	-0.1096	
37	C	0.1095	-0.0083	-0.0171	-0.0412	-0.0973	
38	C	0.1170	-0.0074	-0.0379	-0.0909	-0.1458	
39	C	0.0587	-0.0209	-0.0063	-0.0456	-0.0849	
40	C	0.0353	-0.0059	-0.0129	-0.0265	-0.0616	
41	C	-0.0392	-0.0084	-0.0156	-0.0226	-0.0404	
42	C	0.0602	-0.0167	0.0033	-0.0249	-0.0551	
43	C	0.0960	-0.0072	-0.0141	-0.0343	-0.0804	
44	C	0.0548	-0.0080	-0.0325	-0.0794	-0.1463	
45	C	-0.0231	-0.0038	-0.0081	-0.0267	-0.0656	
46	C	-0.0590	-0.0154	0.0058	0.0012	0.0180	
47	C	-0.1747	-0.0144	-0.0178	-0.0229	-0.0231	
48	C	-0.0508	-0.0129	-0.0175	-0.0201	-0.0137	
49	C	0.0508	-0.0022	-0.0096	-0.0342	-0.0812	
50	C	0.0209	-0.0123	-0.0147	-0.0181	-0.0160	
51	C	-0.0702	-0.0118	0.0085	0.0026	0.0129	
52	C	0.0024	-0.0123	-0.0197	-0.0213	-0.0230	
53	C	0.0973	-0.0039	-0.0160	-0.0473	-0.1058	
54	C	-0.0785	-0.0118	0.0100	-0.0002	0.0021	
55	C	0.0156	-0.0107	-0.0109	-0.0176	-0.0174	
56	C	0.0624	-0.0162	-0.0215	-0.0253	-0.0328	
57	C	0.0736	-0.0083	-0.0157	-0.0167	-0.0108	
58	C	-0.1218	-0.0087	0.0073	0.0069	0.0163	
59	C	0.0678	-0.0083	-0.0158	-0.0167	-0.0112	
60	C	0.0695	-0.0082	-0.0157	-0.0166	-0.0104	
61	C	-0.1008	-0.0141	-0.0195	-0.0226	-0.0352	
62	C	0.0206	-0.0107	-0.0110	-0.0174	-0.0188	
63	C	-0.0038	-0.0103	0.0117	0.0094	0.0444	
64	C	-0.0108	-0.0149	-0.0245	-0.0259	-0.0435	
65	C	-0.0302	-0.0038	-0.0084	-0.0267	-0.0617	
66	C	-0.0535	-0.0129	-0.0177	-0.0203	-0.0154	
67	C	-0.0557	-0.0154	0.0056	0.0010	0.0162	
68	C	-0.1759	-0.0144	-0.0179	-0.0230	-0.0270	
69	C	0.0204	-0.0124	-0.0148	-0.0181	-0.0165	
70	C	0.0042	-0.0123	-0.0197	-0.0213	-0.0229	
71	C	-0.0702	-0.0118	0.0083	0.0026	0.0146	
72	C	0.0612	-0.0163	-0.0215	-0.0253	-0.0345	
73	C	0.0391	-0.0098	-0.0195	-0.0204	-0.0291	

74	C	0.0386	-0.0099	-0.0195	-0.0204	-0.0287	
75	C	-0.1209	-0.0049	0.0116	0.0111	0.0342	
76	C	0.0351	-0.0098	-0.0195	-0.0203	-0.0291	
77	C	0.0054	-0.0124	-0.0196	-0.0213	-0.0234	
78	C	-0.0376	-0.0083	-0.0154	-0.0225	-0.0416	
79	C	-0.0686	-0.0117	0.0084	0.0026	0.0153	
80	C	0.0190	-0.0123	-0.0147	-0.0180	-0.0163	
81	Sc	0.4269	0.3785	0.5106	1.0990	1.8638	1.87E+00
82	Sc	0.4286	0.3785	0.5107	1.0991	1.8637	1.87E+00
83	Sc	0.4259	0.3780	0.5101	1.0986	1.8643	1.86E+00
84	N	-1.3713	-0.2837	-0.5627	-1.2350	-2.1653	-1.82E+00
	F1(B39)	0.0899	-0.8512	-0.9692	-2.0622	-3.4265	0.0000
	F2(SC3N)	-0.0899	0.8512	0.9687	2.0617	3.4265	3.7720
	Total	0.0000	0.0000	-0.0005	-0.0005	0.0000	3.7720

Sc+N calibration curve constraction:

Charges on Sc3N	Sc 1s orbital energies	N 1s orbital energies
-2	-161.91662	-14.30738
-1	-161.95064	-14.31969
0	-161.96970	-14.34887
1	-161.96222	-14.34855
2	-162.03197	-14.3908

Orbital energies in Sc3N@C80 complex	
Sc 1s	-161.9945
N 1s	-14.37341

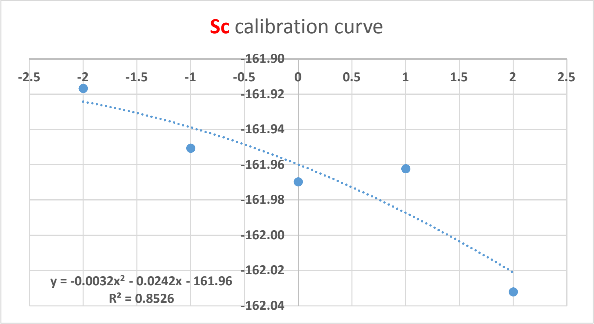

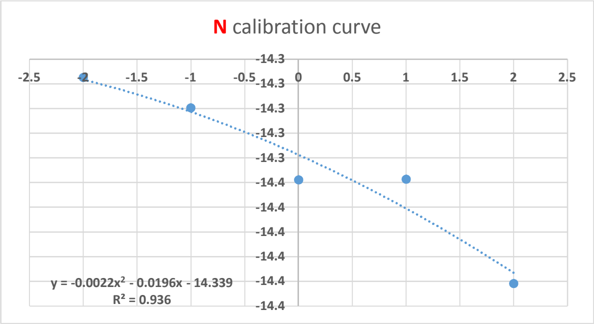

$Q_{predicted}^{Sc} = 1.226$

$Q_{predicted}^{N} = 1.502$

References:

1. Stasyuk, A. J. & Solà, M. Does the endohedral borospherene supersalt $FLi_2@B_{39}$ maintain the "super" properties of its subunits? *Phys. Chem. Chem. Phys.* **19**, 21276-21281, doi:10.1039/C7CP02550E (2017).
2. Weigend, F. & Ahlrichs, R. Balanced basis sets of split valence, triple zeta valence and quadruple zeta valence quality for H to Rn: Design and assessment of accuracy. *Phys. Chem. Chem. Phys.* **7**, 3297-3305, doi:10.1039/B508541A (2005).
3. Weigend, F. Accurate Coulomb-fitting basis sets for H to Rn. *Phys. Chem. Chem. Phys.* **8**, 1057-1065, doi:10.1039/B515623H (2006).
4. M. J. Frisch, G. W. Trucks, H. B. Schlegel, G. E. Scuseria, M. A. Robb, J. R. Cheeseman, G. Scalmani, V. Barone, G. A. Petersson, H. Nakatsuji, X. Li, M. Caricato, A. V. Marenich, J. Bloino, B. G. Janesko, R. Gomperts, B. Mennucci, H. P. Hratchian, J. V. Ortiz, A. F. Izmaylov, J. L. Sonnenberg, Williams, F. Ding, F. Lipparini, F. Egidi, J. Goings, B. Peng, A. Petrone, T. Henderson, D. Ranasinghe, V. G. Zakrzewski, J. Gao, N. Rega, G. Zheng, W. Liang, M. Hada, M. Ehara, K. Toyota, R. Fukuda, J. Hasegawa, M. Ishida, T. Nakajima, Y. Honda, O. Kitao, H. Nakai, T. Vreven, K. Throssell, J. A. Montgomery Jr., J. E. Peralta, F. Ogliaro, M. J. Bearpark, J. J. Heyd, E. N. Brothers, K. N. Kudin, V. N. Staroverov, T. A. Keith, R. Kobayashi, J. Normand, K. Raghavachari, A. P. Rendell, J. C. Burant, S. S. Iyengar, J. Tomasi, M. Cossi, J. M. Millam, M. Klene, C. Adamo, R. Cammi, J. W. Ochterski, R. L. Martin, K. Morokuma, O. Farkas, J. B. Foresman and D. J. Fox, Gaussian 09, revision E.01; Gaussian, Inc.: Wallingford, CT, 2009.
5. Mulliken, R. S. Electronic Population Analysis on LCAO–MO Molecular Wave Functions. I. *J. Chem. Phys.* **23**, 1833-1840, doi:10.1063/1.1740588 (1955).
6. Mulliken, R. S. Electronic Population Analysis on LCAO–MO Molecular Wave Functions. II. Overlap Populations, Bond Orders, and Covalent Bond Energies. *J. Chem. Phys.* **23**, 1841-1846, doi:10.1063/1.1740589 (1955).
7. Löwdin, P. O. On the Non-Orthogonality Problem Connected with the Use of Atomic Wave Functions in the Theory of Molecules and Crystals. *J. Chem. Phys.* **18**, 365-375, doi:10.1063/1.1747632 (1950).
8. Hirshfeld, F. L. Bonded-atom fragments for describing molecular charge densities. *Theor. Chim. Acta* **44**, 129-138, doi:10.1007/bf00549096 (1977).
9. Ritchie, J. P. & Bachrach, S. M. Some methods and applications of electron density distribution analysis. *J. Comput. Chem.*, **8**, 499-509, doi:10.1002/jcc.540080430 (1987).
10. Marenich, A. V., Jerome, S. V., Cramer, C. J. & Truhlar, D. G. Charge Model 5: An Extension of Hirshfeld Population Analysis for the Accurate Description of Molecular Interactions in Gaseous and Condensed Phases. *J. Chem. Theory Comput.* **8**, 527-541, doi:10.1021/ct200866d (2012).
11. Bader, R. F. W. *Atoms in Molecules: A Quantum Theory* (Oxford, 1990).
12. Bader, R. F. W. A quantum theory of molecular structure and its applications. *Chem. Rev.* **91**, 893-928, doi:10.1021/cr00005a013 (1991).
13. AIMAll (Version 14.06.21), Todd A. Keith, TK Gristmill Software, Overland Park KS, USA, 2014 (aim.tkgristmill.com)
14. NBO 6.0. E. D. Glendening, J. K. Badenhoop, A. E. Reed, J. E. Carpenter, J. A. Bohmann, C. M. Morales, C. R. Landis, and F. Weinhold (Theoretical Chemistry Institute, University of Wisconsin, Madison, WI, 2013); http://nbo6.chem.wisc.edu/
